# Construction of a Hierarchical Gene Regulatory Network to Reveal the Drought Tolerance Mechanism of Shanxin Poplar

**DOI:** 10.3390/ijms24010384

**Published:** 2022-12-26

**Authors:** Pengyu Wang, Jingxin Wang, Xiaomeng Sun, Xue Yang, Shilin Sun, Xue Han, Dandan Li, Yucheng Wang

**Affiliations:** 1 College of Forestry, Shenyang Agricultural University, Shenyang 110866, China; 2 Key Laboratory of Forest Tree Genetics, Breeding and Cultivation of Liaoning Province, Shenyang 110866, China

**Keywords:** gene regulatory network (GRN), drought stress, Shanxin poplar (*Populus davidiana* × *P. bolleana*), transcription factor, TF-DNA interactions, PdbERF3

## Abstract

Drought stress is a common adverse environment that plants encounter, and many drought-tolerant genes have been characterized. The gene regulatory network (GRN) is important in revealing the drought tolerance mechanism. Here, to investigate the regulatory mechanism of Shanxin poplar (*Populus davidiana* × *P. bolleana*) responding to drought stress, a three-layered GRN was built, and the regulatory relationship between genes in the GRN were predicted from expression correlation using a partial correlation coefficient-based algorithm. The GRN contains 1869 regulatory relationships, and includes 11 and 19 transcription factors (TFs) in the first and second layers, respectively, and 158 structural genes in the bottom layers involved in eight enriched biological processes. ChIP-PCR and qRT-PCR based on transient transformation were performed to validate the reliability of the GRN. About 88.0% of predicted interactions between the first and second layers, and 82.0% of predicted interactions between the second and third layers were correct, suggesting that the GRN is reliable. Six TFs were randomly selected from the top layer for characterizing their function in drought, and all of these TFs can confer drought tolerance. The important biological processes related to drought tolerance were identified, including “response to jasmonic acid”, “response to oxidative stress”, and “response to osmotic stress”. In this GRN, PdbERF3 is predicted to play an important role in drought tolerance. Our data revealed the key regulators, TF-DNA interactions, and the main biological processes involved in adaption of drought stress in Shanxin poplar.

## 1. Introduction

Drought is the main adverse environment that plants encounter, which induces an imbalance between root water uptake and water loss via transpiration, leading to plant dehydration. In addition, drought severely affects the survival, growth, and productivity of plants. More than 38% of the world’s population are living in the drylands, which are the most sensitive areas to human activities and climate change. In addition, it has been estimated that the drylands will cover half of the global land surface by the end of this century. Therefore, it is necessary to study the mechanism of drought tolerance of plants [1]. 

In order to adapt to adverse environments, plants employ a series of strategies at the developmental, physiological, biochemical, and molecular levels [2]. Plants respond to drought stress by regulating the expression of a series of genes to form a gene regulatory network (GRN). GRNs are constituted of a series of genes, including a few high-level regulators constituted by transcription factors (TFs), some middle-level regulators that are also TFs, and many structural genes that constitute the bottom layer. In a GRN, high-level TFs serve as key receptors of the global modulators, playing roles in activating the network or initiating a coordinated process, and also regulate the middle-level TFs that, in turn, control the structural genes at the bottom layer that directly implement the specific processes. Therefore, TFs, especially the TFs in the first level, play regulatory roles, and are of central importance. 

Recently, some algorithms have been developed to build the gene regulatory network, such as Bayesian networks (BNs) [3], the Algorithm for the Reconstruction of Accurate Cellular Networks (ARACNE) [4], the bottom-up graphical Gaussian model (bottom-up GGM) algorithm [5], the backward elimination random forest (BWERF) algorithm [6], and triple-gene mutual interaction (TGMI) [7], dynGENIE3 [8], and GRNBoost2 and Arboreto [9]. 

Some GRNs have been built that were mainly according to gene expression and yeast one hybrid (Y1H). For instance, based on the specific expression of genes differentially expressed between female and male flowers, a gene regulatory network with the core of CpAGL11-CpSUPL-CpHEC2 functioning was constructed. In this GRN, several genes involved in auxin/brassinosteroid signal transduction were revealed, and the brassinosteroid biosynthesis-related genes associated with female and male flowers at different developmental stages were identified [10]. To investigate the regulatory mechanism of *Betula platyphylla* in response to drought stress, Jia et al. [11] constructed a three-layer hierarchical GRN using a partial correlation coefficient-based algorithm. This GRN includes 68 transcription factors (TFs) and 252 structural genes with 1448 predicted regulatory relationships. This GRN identified that BpERF017, BpNAC090, and BpAGL61 are key regulators in drought tolerance. Chen et al. [12] built a GRN in birch under cold stress conditions, in which six mitogen-activated protein kinases were linked, and the MEKK1-MKK2-MPK4 cascade in response to cold was presented. A global *Fusarium graminearum* GRN was built, the connectivity between key regulators and their target genes was revealed, and some unique functions, such as DNA replication, cell cycle, translation, transcription, and stress responses were enriched [13]. Some GRNs were built using microarray data. A GRN involved in an intricate core oxidative stress regulatory network was built based on an *A. thaliana* microarray expression array, and the TFs, including WRKY6, ERF6, NAC032, NAC13, and NAC053, interact and play roles in detoxification [14]. 

In addition, some GRNs were built mainly according to ChIP and qRT-PCR data. For instance, a four-layer GRN directed by PtrSND1-B1 was constructed, and PtrSND1-B1 was found to direct 57 TF-DNA interactions, linking 17 TFs to regulate 27 genes involved in cell wall biosynthesis [15]. 

Some GRNs were built using yeast one hybrid (Y1H). For instance, Shi et al. [16] built a GRN between mycorrhizal symbiosis-related genes and TFs using Y1H technology. This GRN is governed by the conserved P-sensing pathway, and is centered on the TFs involved in the phosphate starvation response (PHR). Phosphate starvation responses are needed for the regulation of symbiosis-related genes via the P1BS motif and mycorrhizal symbiosis. 

Poplar ‘Shanxin’ (*Populus davidiana* × *P. bolleana*) is an important plantation species of poplar that is widely distributed in Northeast China, and has the advantages of growing fast, strong adaptability, and high drought stress tolerance. 

In the present study, we investigate the response of Shanxin poplar to drought stress on a systems level. The differentially expressed genes (DEGs) in response to drought stress for 1, 3, 5, 9, 12, and 24 h were identified, and a three-layer hierarchical GRN was built, where the regulatory interaction was predicted based on expression correlation. The key regulators of drought tolerance were identified, and the interaction between genes responding to drought was revealed. This GRN provides necessary data for revealing drought tolerance mechanisms of poplar.

## 2. Results

### 2.1. Dynamic Changes of Poplar Transcriptome in Response to Drought Stress

Time-course cDNA libraries in response to drought stress were constructed to explore the transcriptional changes of Shanxin poplar. A total of 7 stress time points (including 1 control for all treatments) were included. Each treatment contained 3 independent biological replicates, and a total of 21 cDNA libraries were constructed and sequenced. In total, 5.75 Gb clean reads for the 21 libraries were yielded (Table S1). Of the reads, 81.99% to 90.70% could be mapped to the genome of Shanxin poplar (Table S2). The differentially expressed genes (DEGs) between the control samples and each of the treated time points were identified. There were 247, 504, 716, 228, 1405, and 1115 DEGs identified after drought stress for 1, 3, 5, 9, 12, and 24 h, respectively (Table S3). The expression profiles of genes in the GRN were shown as a heatmap (Figure S1). In this heatmap, the expression of genes could be classified into three main patterns. Pattern 1 presented the expression profile of genes that were highly induced at stress for 1 and 5 h, but was highly decreased at stress for 9 and 24 h. Pattern 2 showed the expression profile that the expression of genes was highly decreased at stress for 1, 3, and 5 h, but was highly increased at stress for 12 and 24 h. Pattern 3 is the expression profile that the expression of genes was highly induced at stress for 0, 3, and 9 h, but was highly decreased at stress for 1 and 5 h. 

### 2.2. Construction of Gene Regulatory Network (GRN) in Response to Drought Stress

We analyzed these DEGs with Gene Ontology (GO) annotation in the biological process (Figure S2 and Table S4). To identify the regulatory relationships of these DEGs, we constructed a GRN using the basic partial correlation algorithm. A three-layer GRN was constructed containing 1869 regulatory relationships, including 11 TFs in the first layer, 19 TFs in the second layer, and 158 structural genes in the bottom layer (for gene name, see Table S5). Out of 11 TFs, 6 were randomly selected to further verify whether their expression is a response to drought stress using qRT-PCR, and the result indicated that the expressions of these genes are all induced by drought stress (Figure S3). The 158 structural genes were classified into 8 GO terms (Table S6). 

**Figure 1:**
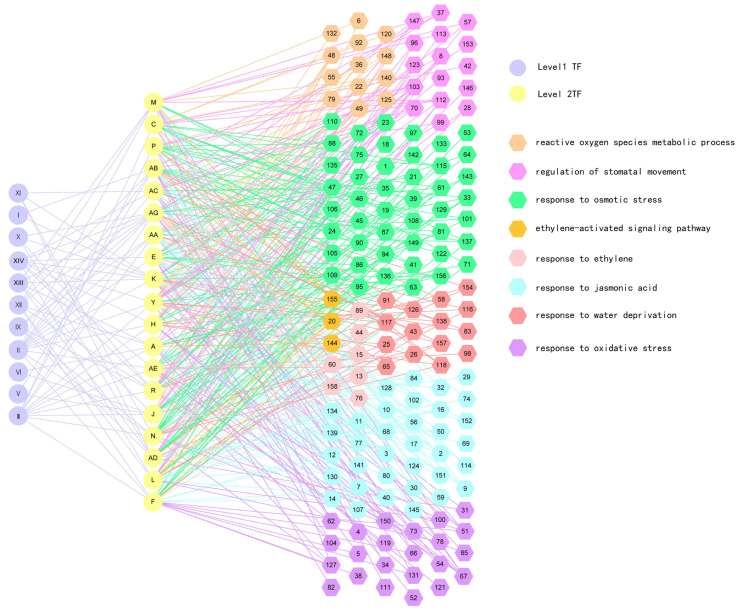
The gene regulatory network (GRN) of *Populus davidiana* × *P. bolleana* in response to PEG-induced drought stress. A three-layer GRN was constructed. The first layer contains 11 transcription factors (TFs) numbered I: PdbCCCH29 (GenBank Number: OP434351), II: PdbERF3 (GenBank Number: OP434346), III: PdbSCL1 (GenBank Number: OP434347), V: PdbZAT10 (GenBank Number: OP434352), VI: PdbATHB7 (GenBank Number: OP434348), IX: PdbHK4 (GenBank Number: OP434350), X: PdbTIFY9 (GenBank Number: OP434353), XI: PdbHK3 (GenBank Number: OP434349), XII: PdbABF3 (GenBank Number: OP434354), XIII: PdbMYB102 (GenBank Number: OP434355), and XIV: PdbAL5 (GenBank Number: OP434356). The second layer contains 19 TFs, and the bottom layer contains 158 structural genes that are involved in 8 enriched GO biological processes involved in drought tolerance. This GRN was constructed in a drought stress time-series experiment with 6 time points: 1, 3, 5, 9, 12, and 24 h. The numbers representing the gene names in GRN are shown in Table S5.

In total, 1869 predicted interactions between genes were included, including 65 interactions between the TFs in the first and second layers, and 1804 interactions between the TFs in the second layer and structural genes in the third layer. All these interactions lead to the TFs in the first layer regulating the structural genes in the bottom layer by regulating the TFs in the second layer. These interactions led to the eight GO terms being activated, including “reactive oxygen species metabolic process”, “ethylene-activated signaling pathway”, “regulation of stomatal movement”, “response to jasmonic acid”, “response to water deprivation”, “response to ethylene”, “response to oxidative stress”, and “response to osmotic stress” (Figure 1). As all these eight biological processes are found to be involved in drought stress according to previous studies, these eight GO terms may play a main role in drought tolerance in Shanxin poplar, and the regulators in the first layer of the GRN might be the key regulators in regulating drought tolerance of Shanxin poplar. One of the specific features is that the highest percentage of genes in the GO term is “response to osmotic stress”, which is consistent with the feature of the GRN in response to drought.

### 2.3. Validation of the Regulatory Relationship between First and Second Layers in GRN

**Figure 2:**
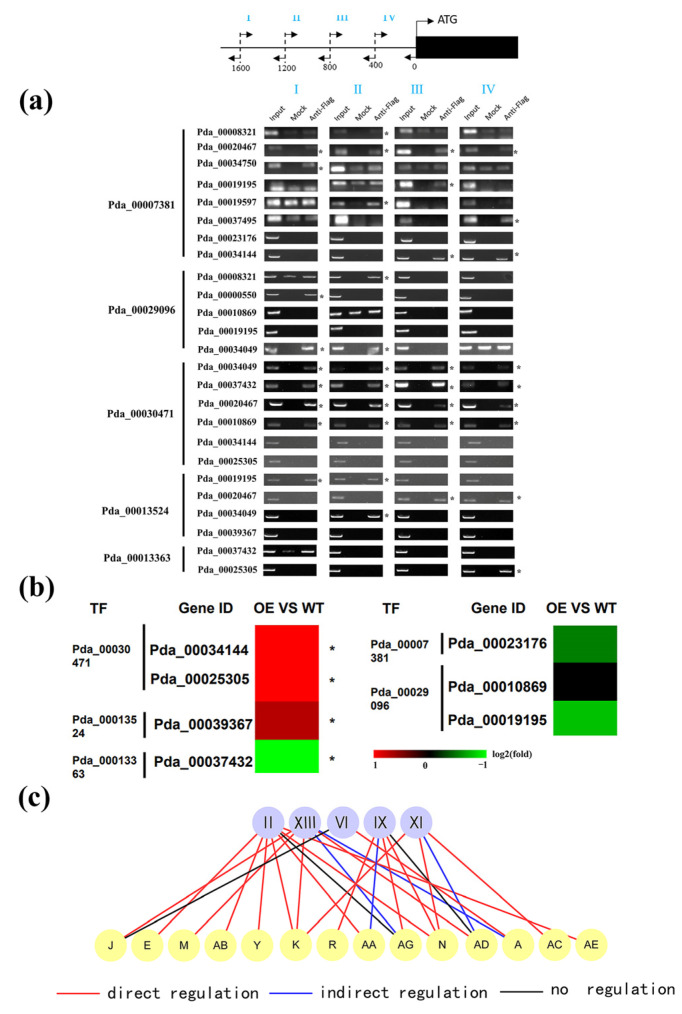
Verification of the regulatory relationship between the first and second layers in the GRN. (**a**) Verification of the direct regulatory relationships of the TFs between the first and the second layers using ChIP-PCR. The promoters (−2000 to 0 bp) were divided into four equal-length fragments (I to IV) for ChIP-PCR (right panel). Input, mock, and anti-FLAG indicate the chromatin before immunoprecipitation (IP), IP with no antibody (mock), and IP with anti-FLAG antibody (anti-FLAG), respectively. An asterisk (*) indicates the binding of TF to the truncated promoters. (**b**) Verification of indirect regulatory relationship using qRT-PCR. The heatmap represents the relative expression (fold change) of the genes in transiently transformed poplar plants overexpressing each TF relative to the control. Red indicates up-regulation; green indicates down-regulation. An asterisk (*) indicates significant regulation (*p* < 0.05). (**c**) A summary of the regulatory relationship between the first and second layers in the GRN. The gene names are shown in Table S5.

In a direct relationship, transcription factors bind to the gene’s promoter to either increase or decrease the expression of the gene, whereas in an indirect relationship, transcription factors do not directly bind to the gene’s promoter but instead affect the gene’s expression by directly regulating other TFs. For identification of a regulatory relationship between the first and second layers, 5 TFs were randomly selected from 11 TFs in the first layer. The truncated promoters (2000 bp in length) of the putative target genes were divided into four equal regions for ChIP-PCR analysis. The gene interactions of the selected TFs were randomly selected. Among the 25 studied interactions, 18 interactions were found that the TFs can bind to the promoters of target genes (Figure 2a).

According to the ChIP-PCR result, there were seven interactions that were not the direct regulatory relationship among the 25 studied interactions. To determine whether these interactions are indirect regulatory relationships or not, qRT-PCR was performed to compare the gene expression between plants overexpressing TF and the control plants. The results showed that four out of the seven studied interactions have an indirect regulatory relationship (fold change > 2; *p* < 0.05), and the other three predicted interactions had no interaction (Figure 2b). Taken together, we summarized that 72.0% of predicted interactions are direct regulatory relationships, and 16.0% of predicted interactions are indirect regulatory relationships (Figure 2c). In total, 88.0% of predicted interactions in the GRN are true. 

### 2.4. Verification of the Regulatory Relationship between the Second and Third Layers 

**Figure 3:**
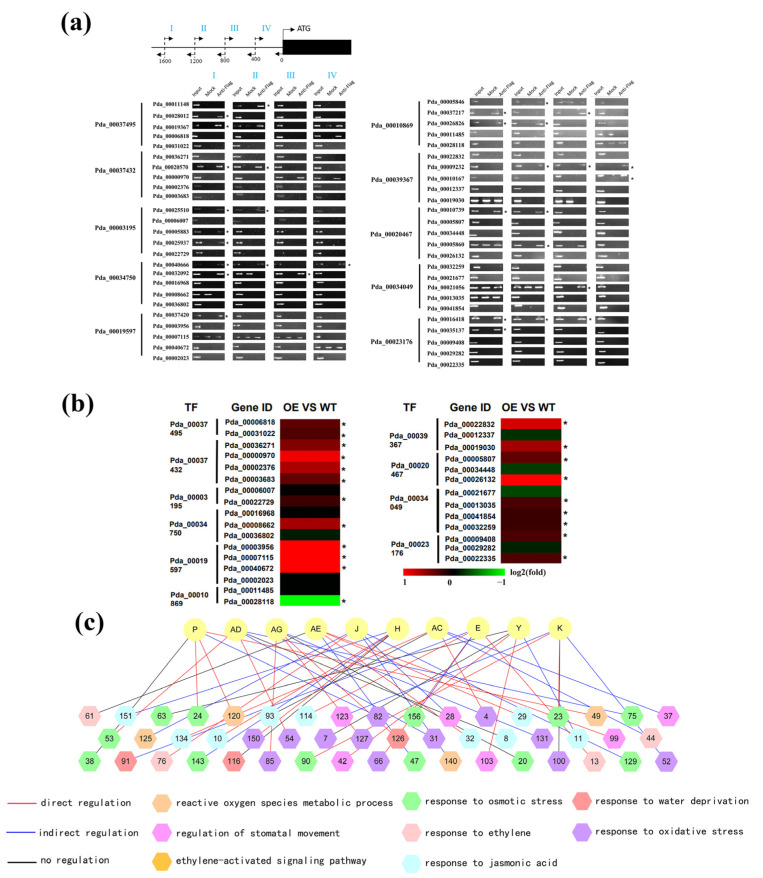
Verification of the regulatory relationship between the second and third layers in the GRN. (**a**) Verification of the regulatory relationships between the second and third layers using ChIP-PCR. The promoters (−2000 to 0 bp) were divided into four equal-length fragments (I to IV) for ChIP-PCR. Input, mock, and anti-FLAG indicate the chromatin before immunoprecipitation (IP), IP with no antibody (mock), and IP using anti-FLAG antibodies (anti-FLAG), respectively. An asterisk (*) indicates the binding of a TF to the truncated promoters. (**b**) Verification of the indirect regulatory relationship between the second and third layers using qRT-PCR. The heatmap represents the relative expression (fold change) of the genes in poplar plants overexpressing each TF relative to the control (transformed with empty vector). Red indicates up-regulation; green indicates down-regulation. An asterisk (*) indicates significant gene expression regulation (*p* < 0.05). (**c**) A summary of the regulatory relationship between the second and third layers in the GRN. The gene names are shown in Table S5.

We next analyzed the reliability of the regulatory relationship between the TFs in the second layer and genes in the third layer of the GRN. ChIP-PCR was first performed to determine whether there was direct interaction among these interactions. In total, 10 TFs were randomly selected, and 50 putative regulatory relationships were randomly selected for testing. According to ChIP-PCR results, there were 20 putative direct interactions among the 50 studied interactions (Figure 3a). The other 30 putative interactions did not show direct interaction, and qRT-PCR was further performed to study whether these interactions are indirect. The results showed that there were 21 interactions among the 30 remaining predicted interactions, which were indirect regulatory relationships (Figure 3b). Taken together, 40.0% of interactions showed direct regulation, and about 42.0% showed indirect regulation (Figure 3c). Therefore, 82.0% of predicted interactions in the GRN were true interactions. 

### 2.5. Determination of Drought Tolerance of the Regulator Genes in the First Layer of GRN

**Figure 4:**
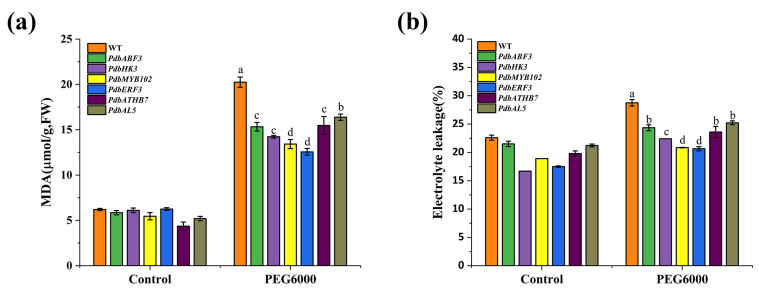

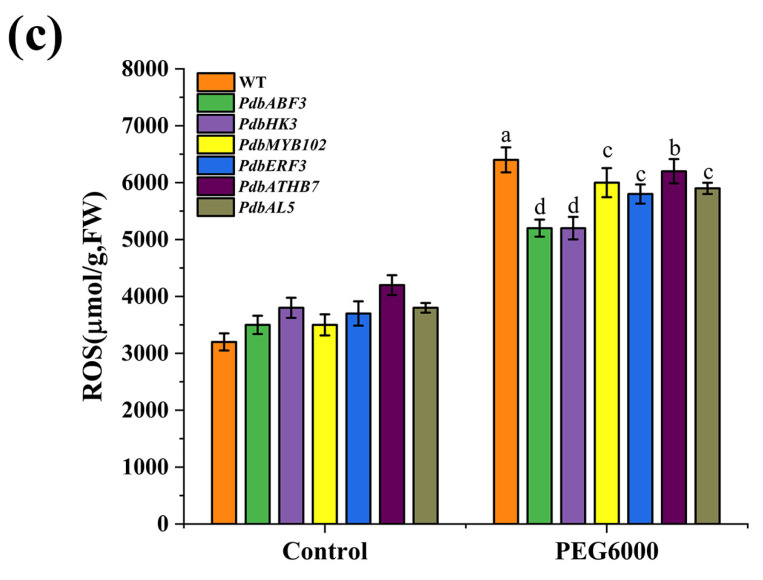
Determination of drought tolerance properties of the first-layer regulators of the GRN (**a**) MDA content analysis; (**b**) electrolyte leakage assay; (**c**) ROS content analysis. The genes were transiently transformed into *P. davidiana* × *P. bolleana* plants for overexpression; the plants transiently transformed with empty vector were used as controls. At 48 h after transformation, the plants were watered with 20% PEG6000 solution on roots for 24 h. The data with different letters indicate significant differences (*p* < 0.05). As the transformations were performed in different batches, each batch had an independent control. The gene names and GenBank numbers are *PdbERF3* (GenBank Number: OP434346), *PdbABF3* (GenBank Number: OP434354), *PdbMYB102* (GenBank Number: OP434355), *PdbATHB7* (GenBank Number: OP434348), *PdbHK3* (GenBank Number: OP434349), and *PdbAL5* (GenBank Number: OP434356).

As the regulators in the first layer play important roles in gene expression regulation, we randomly selected six TFs from the first layer to study whether they could confer drought tolerance. These six TFs were cloned into gene expression vectors and transiently expressed in Shanxin poplar. After transformation for 48 h, the physiological traits involving abiotic stress tolerance, including electrolyte leakage, malondialdehyde (MDA), and reactive oxygen species (ROS) content, were investigated. Under normal conditions, the MDA content, electrolyte leakage, and ROS content were generally similar among all the studied plants (Figure 4). However, under Polyethylene glycol (PEG) stress conditions, all the poplar plants overexpressing these six TFs showed significantly reduced (*p* < 0.05) MDA content (Figure 4a), electrolyte leakage rate (Figure 4b), and ROS content (Figure 4c) compared with the control plants (transient expression of empty pROKII). This result indicated that these six TFs are all involved in drought stress, and could confer drought tolerance to poplar. At the same time, we determined whether the TFs in the first layer can induce the expression of the second and third layers. The 6 TFs were transiently expressed in Shanxin poplar, and 18 genes in the second layer and 18 genes in the third layer were randomly selected, and their expression was analyzed using qRT-PCR. The results indicated that all the studied genes were induced by these six TFs (Figure S4), further confirming that the GRN is reliable. As these TFs are in the top layer of the GRN and play roles in drought tolerance regulation, the capability of conferring drought tolerance further supports the reliability of the GRN.

## 3. Discussion

In the present study, we constructed a three-layer GRN to investigate the drought response mechanism in Shanxin poplar. In this dynamic GRN, the first and second layers comprised TFs, while the third layer only included structural genes. In general, the first-layer genes served as the global modulators, and the TFs in the second layer acted as master-like roles. The genes in the third layer served as the executors. The regulatory relationship among the genes was revealed in this GRN, and the key regulators were identified according to the regulatory relationship of the genes. In addition, ChIP-PCR and qRT-PCR were used to study the reliability of the GRN according to the method of Jia et al. [11], and we found that more than 80.0% of the interactions predicted in the GRN were true, suggesting that this GRN is useful in revealing the drought tolerance mechanism of Shanxin poplar.

### 3.1. PdbERF3 Is the Most Important Regulator in Response to Drought Stress

The ethylene-responsive factors (AP2/ERF) play vital roles in regulating biotic and abiotic stress, plant growth, and development [17,18,19]. The AP2/ERF family can be classified into the ERF, AP2, Soloist, and RAV sub-families. The ERF sub-family members are generally involved in abiotic stress, pathogen stress responses, and the ethylene signaling pathway, and are involved in the regulation of genes by binding to the GCC-Box (sequence AGCCGCC) motif [20]. ERF TFs play important roles in mediating abiotic stress tolerance, and several ERF TFs have been found to be viable candidates for improving abiotic stress tolerance in plants, such as TaERF1 [21], PtERF109 [22], and BpERF11 [23].

In the present study, we found that ERF3 is the upstream regulator of the GRN, which can regulate 12 TFs in the middle layer. In addition, these 12 TFs regulate all the structural genes, which are involved in 1134 interactions with the genes in the third-layer regulation relationships, accounting for 63.0% of the total interactions between the second and third layers (Figure 1). Furthermore, ERF3 (GenBank Number: OP434346) can confer drought tolerance, and the plants overexpressing ERF3 showed the lowest electrolyte leakage and MDA content among the studied TFs (Figure 4). These results indicated that ERF3 can indirectly regulate all the structural genes in the GRN, suggesting that it plays a very important role in drought tolerance response.

### 3.2. Hormone Signaling Plays an Important Role in Drought Stress Response

Plants can react quickly and specifically to variable environmental conditions by producing phytohormones. Jasmonic acid (JA) is a hormone that is important for plants, which is not only involved in plant development, but also associated with the defense responses against pathogenic attacks and adverse environments such as drought, cold, salinity, heat, heavy metals, elevated ozone, water logging, and UV radiation [24,25,26,27]).

The phytohormone ethylene is found to be produced in response to variable abiotic stresses [28]. Ethylene signaling is found to be indispensable for plant rapid response and tolerance to abiotic stress [29]. In the present study, the identification of DEGs showed that the genes belonging to “response to jasmonic acid”, “response to ethylene”, and “ethylene-activated signaling pathway” were significantly enriched, and no other GO terms involved in signaling pathways were enriched. In addition, there were 45 genes belonging to these three GO terms in the GRN, accounting for 28.5% of total structural genes. These results indicate that the JA and ethylene signaling pathways are very important for drought responses in Shanxin poplar. 

### 3.3. Stomatal Movements Play a Role in Drought Tolerance

The regulation of stomatal pore aperture plays a key role in plant drought tolerance and resilience [30]. To adjust to lower soil moisture content, a decrease in transpiration caused by partial or total stomatal closure is connected with changes in leaf water status [31]. In the present study, there were 16 genes involved in stomatal movement in the constructed GRN, such as sodium/hydrogen exchanger 2 (NHX2), two-pore calcium channel protein 1 (TPC1), and jasmonoyl-L-amino acid synthetase (JAR1). Several genes involved in stomatal movement were enriched under drought stress, suggesting that one of the main mechanisms for drought tolerance is the control of stomatal closure in Shanxin poplar. 

### 3.4. Scavenging ROS Is Necessary for a Drought Response in Shanxin Poplar

Plants rapidly generate ROS when exposed to adverse environments, such as drought, high salinity, cold, or heat. An excess of ROS causes oxidative damage to cell components, such as lipids, proteins, nucleic acids, and carbohydrates. In addition, high amounts of ROS will disturb the cellular membrane and the synthesis of proteins, leading to damage to cells and tissue [32]. However, low amounts of ROS act as signaling molecules to regulate physiological and biological processes [33], and also serve as a signal transduction mechanism for environmental adaptation [34]. Previous studies showed that the genes/proteins involved in “reactive oxygen species metabolic process” were highly enriched to scavenge excess ROS [35,36], and drought stress is found to mainly promote the energy metabolism pathway, such as the reactive oxygen species metabolic process to adapt drought stress [35]. Therefore, scavenging excess ROS and modulation of ROS to suitable levels are critical for the stress tolerance of plants [37,38,39,40]. 

In the present study, our results showed that there were 23 and 13 genes, respectively, involved in “response to oxidative stress” and “reactive oxygen species metabolic process”, whose total accounts for 22.90% of total structural genes. Such a high percentage of genes in the GRN is involved in ROS response, suggesting that drought stress induced a high level of ROS, and scavenging ROS plays an important role in drought adaption in Shanxin poplar.

Previous studies showed that both the GO terms of “response to water deprivation” and “response to osmotic stress” were found to be most obviously linked to drought stress, and the genes involved in these two processes were highly enriched by drought stress [41,42]. In the present study, the genes involved in “response to osmotic stress” accounted for 29.30% of total genes in the GRN, which is the largest GO term. At the same time, the genes in “response to water deprivation” accounted for 9.60% of genes in the GRN. Therefore, these two GO terms constituted a main part in the GRN (nearly 40.0% genes of GRN), suggesting that “response to water deprivation” and “response to osmotic stress” are the main molecular responses to drought stress, and may play quite important roles in drought adaption in Shanxin poplar.

## 4. Materials and Methods

### 4.1. Plant Materials and Drought Treatments 

The Shanxin poplar (*Populus. davidiana* × *P. bolleana*) was collected in the “Key Laboratory of Forest Tree Genetics, Breeding and Cultivation of Liaoning Province”, which was generated from the female parent *P. davidiana*, and the male parent of *P. bolleana* by Heilongjiang Provincial Shelterbelt Institute in China. The plantlets of *P. davidiana × P. bolleana* were grown in pots with a mixture substance of perlite/soil (2:1 *v*/*v*) in a greenhouse (20–24 °C, 16 h light/8 h dark photoperiod, and relative humidity of 70.0%). The well-watered plants growing for three weeks were treated with 20% (*w*/*v*) polyethylene glycol (PEG)6000 solution (Sigma–Aldrich, St. Louis, MO, USA) on their roots. The plants were treated with PEG6000 for 0, 1, 3, 5, 9, 12, and 24 h, respectively. The treatment times were set so that all the samples could be harvested at the same time to reduce the molecular background caused by temporal rhythm. After treatment, the aerial part of the plants was harvested for RNA-seq. Each sample was from at least six different plantlets, and three biological replicates were conducted for each time point. 

### 4.2. Library Preparation and Differential Expression Analysis

RNA concentration and purity were measured using NanoDrop 2000 (Thermo Fisher Scientific, Wilmington, DE, USA). RNA integrity was assessed using the RNA Nano 6000 Assay Kit of the Agilent Bioanalyzer 2100 system (Agilent Technologies, Palo Alto, CA, USA). A total amount of 1 μg total RNA per sample was used as input material for RNA sample preparations. Sequencing libraries were generated using the NEB Next^®^ Ultra™ RNA Library Prep Kit for Illumina^®^ (NEB, USA) following the manufacturer’s recommendations, and index codes were added to attribute sequences to each sample.

The libraries were sequenced on an Illumina platform (Illumina HiSeq 2500), and paired-end reads were generated. Raw data of Fastq format were firstly processed through Fastp 0.21.0 [43]. In this step, clean data were obtained by removing reads containing adapters, reads containing poly-N, and low-quality reads. At the same time, Q20, Q30, GC content, and sequence duplication level of the clean data were calculated. All the downstream analyses were based on clean data of high quality. The reads were mapped to the genome of Shanxin poplar. The genome sequence of *P. davidiana* × *P. bolleana* was submitted to GenBank with the BioProject accession number PRJNA867039 (https://www.ncbi.nlm.nih.gov/bioproject/?term=PRJNA867039) accessed on 8 August 2022.

Differential expression analysis of drought and control was performed using edgeR 3.38.4 [44]. The false discovery rate (FDR) < 0.05 and fold change > 1.5 or <0.66 were set as the threshold for significant differential expression [45]. Gene expression was estimated by fragments per kilobase of transcript per million fragments mapped [46].

### 4.3. Identification of Biological Processes Involved in Drought Stress

Poplar has limited Gene Ontology (GO) annotation; therefore, we annotated the poplar genes using Arabidopsis GO annotations. If multiple BLAST +2.13.0 [47] hits were returned, only the bit score of the best hit was used in the clustering computation with a threshold of E-value < 1 × 10^−5^ [48]. Then, GO enrichment of the DEGs was conducted to determine the enriched biological processes in Shanxin poplar under drought stress conditions. Gene Ontology (GO) enrichment analysis of the DEGs was implemented by the GO-seq R packages based on Wallenius non-central hypergeometric distribution [49]. 

### 4.4. Construction of Multilayered Hierarchical Gene Regulatory Network in Response to Drought Stress

A partial correlation coefficient-based algorithm was employed to build the TF-based GRN according to the method described by Jia et al. [11]. In detail, the algorithm script (Supplementary Script Files S1 and S2) was based on the fact that if a group of genes shared the same profile, they are likely to be co-regulated by the same regulator. The DEGs of all stress times were divided into 2 groups: (1) regulatory genes, such as TF encoding genes; and (2) structural genes. The structural genes enriched in different GO terms served as the genes in the bottom layer to construct the regulatory network. The relationship between TF (from regulatory genes in DEGs) and structural genes was determined by using a partial correlation coefficient-based algorithm. In this algorithm, a pair of co-expressed genes were first identified, which were termed x and y, and a TF termed z. If Pearson’s correlation coefficients of *x* and *y* were significant (*r_xy_* ≥ 0.8 and *p* < 0.001), at the same time, considering the TF (*z*), the partial correlation coefficient of *x* and *y* was non-significant ($r_{xy|z}=\frac{r_{xy}-r_{xz}r_{yz}}{\sqrt{1-r_{xz}^{2}}\sqrt{1-r_{yz}^{2}}}$, *r_xy_*|*z* ≤ 0.3) [50]. Then, the co-expression of x and y was defined, which was derived from their co-regulation by *z*.

### 4.5. Plant Expression Vector Construction and Transient Transformation

For overexpression vector construction, the full-length coding sequence (CDS) of each gene was fused with a 3×FLAG tag and was cloned into the pROKII vector under the control of the 35S promoter. The sequence was further verified by nucleotide sequencing and using cloning. All the primer sequences of primers used to construct these vectors are shown in Table S7. 

Transient transformation was performed following the description of Zang et al. [51]. In brief, the *Agrobacterium tumefaciens* EHA105 cells were grown in LB liquid medium to OD600 of 0.6–0.7 under 180 rpm at 28 °C. The cultures were then centrifuged at 3000× *g* to harvest the *A. tumefaciens* cells, and were adjusted to an OD600 of 0.8 with a transformation solution [1/2 MS, pH 5.8 + 120 μM acetosyringone + 2.5% (*w*/*v*) sucrose + Tween20 (0.01%, *v*/*v*)]. For transient genetic transformation, the plants were soaked in the transformation solution with shaking at 90 rpm for 2.5 h at 25 °C. Following this, the plants were washed with distilled water quickly and were planted vertically on 1/2 MS solid medium [1/2 MS, pH 5.8 + 120 μM acetosyringone + 1% (*w*/*v*) sucrose] for 48 h, and the transformed plants were then used for subsequent treatments and experiments. 

### 4.6. Validation of the Regulatory Relationship in GRN

To verify the regulatory relationships between the TF and TF, or TF and structural gene in the GRN, ChIP-PCR was first performed. TFs were randomly selected from the first layer and second layers of the GRN. The selected TF was fused with an N-terminal 3 × FLAG under the control of the 35S promoter in the pROKII vector, for overexpression, and immunoprecipitation analysis was performed using anti-Flag antibody.

### 4.7. Chromatin Immunoprecipitation (ChIP)

ChIP was performed using the plant of Shanxin populus transiently transformed with 3×FLAG, according to the method of Zhao et al. [52]. In brief, the nucleus was isolated, and the chromatin was sonicated and immunoprecipitated with anti-FLAG antibodies (ChIP+) or rabbit anti-hemagglutinin (HA) antibody (ChIP–, negative control). The reaction system was as follows: 1 µL of ChIP product, 0.5 µM of each forward or reverse primer, and 10 µL of ChamQ Universal SYBR qPCR Master Mix (Vazyme Biotech, Nanjing, China), with a total volume of 20 µL. The thermal profiles were 30 cycles of 94 °C for 30 s, 57 °C for 30 s, and 72 °C for 30 s. The primers used for ChIP-PCR are shown in Table S8.

### 4.8. Real-Time RT-PCR

Total RNA was isolated from each sample using the Total RNA Isolation Kit (BioTeke Corporation, Beijing, China) and was reverse transcribed into complementary DNA (cDNA) using the First Strand cDNA Synthesis kit (Vazyme, Nanjing, China) with oligo-dT primers as the initial primer. Synthesized cDNA was diluted 5-fold using ultra-pure water for qPCR analysis. The qPCR reaction consisted of 0.5 µM of each primer, 5 µL of ChamQ Universal SYBR qPCR Master Mix (Vazyme), and 1 µL of cDNA sample, with a total volume of 10 µL. The qPCR was performed on a qTower 2.2 (Analytik Jena AG, Germany) with the thermal cycles of 94 °C for 30 s, 40 cycles of 94 °C for 30 s, 60 °C for 30 s, and 72 °C for 30 s. The expression was normalized using TUBULIN2 as the internal reference gene, and was calculated using the comparative CT method. Three independent experimental replicates were performed, each consisting of three independent biological replicates. The primers used for qRT-PCR are shown in Table S9.

### 4.9. Physiological Change Analysis 

Characterization of drought tolerance of genes was performed based on transient genetic transformation following the methods described by Zang et al. [51]. After transformation for 48 h, the transformed plants were moved to culture medium containing 20% (*w*/*v*) PEG for 24 h for drought treatment, and the transiently transformed plants growing on normal culture medium were used as the controls. The electrolyte leakage rate was determined according to the method of Dionisio-Sese and Tobita. [53,54]. The malondialdehyde (MDA) content was measured following the description of Rao and Sresty (2000). The reactive oxygen species (ROS) content was detected using a commercially available kit from Nanjing Senbeijia Bioengineering Institute (Nanjing, China). 

### 4.10. Statistical Analysis

The experimental data were statistically analyzed using one-way analysis with repeated measures of variance (one-way ANOVA). Statistical analyses were performed using the Statistical Package for the Social Sciences (SPSS 22, IBM Corp, Armonk, NY, USA), and statistical significance was set at *p* < 0.05.

## 5. Conclusions

In the present study, we constructed a GRN in response to drought stress in Shanxin poplar, and the reliability of this GRN was further confirmed. We identified that eight GO terms play important roles in drought tolerance of Shanxin poplar, and the regulators and gene interactions controlling these eight GO terms were revealed. The key regulators of drought tolerance were identified from this GRN, which are good candidate genes for characterizing drought tolerance of Shanxin poplar. 

## Data Availability

Raw Illumina sequence data were deposited in the National Center for Biotechnology Information (NCBI) and can be accessed in the sequence read archive (SRA) database (https://www.ncbi.nlm.nih.gov/sra, accessed on 8 August 2022). The accession number is listed in Table S1, and this record has been released. The genome sequence of *Populus davidiana* × *P. bolleana* has been submitted to GenBank with the BioProject accession number PRJNA867039 (https://www.ncbi.nlm.nih.gov/bioproject/?term=PRJNA867039) accessed on 8 August 2022.

## Supplementary Materials

The following supporting information can be downloaded at: https://www.mdpi.com/article/10.3390/ijms24010384/s1.
