# Supplementary material for: Construction of a Hierarchical Gene Regulatory Network to Reveal the Drought Tolerance Mechanism of Shanxin Poplar
Source: Int J Mol Sci. 2022 Dec 26;24(1):384. doi: 10.3390/ijms24010384 (PMC9820611; doi:10.3390/ijms24010384)
Supplement: Supplementary file 1 [file ijms-24-00384-s001.zip › Figure S1.pdf]

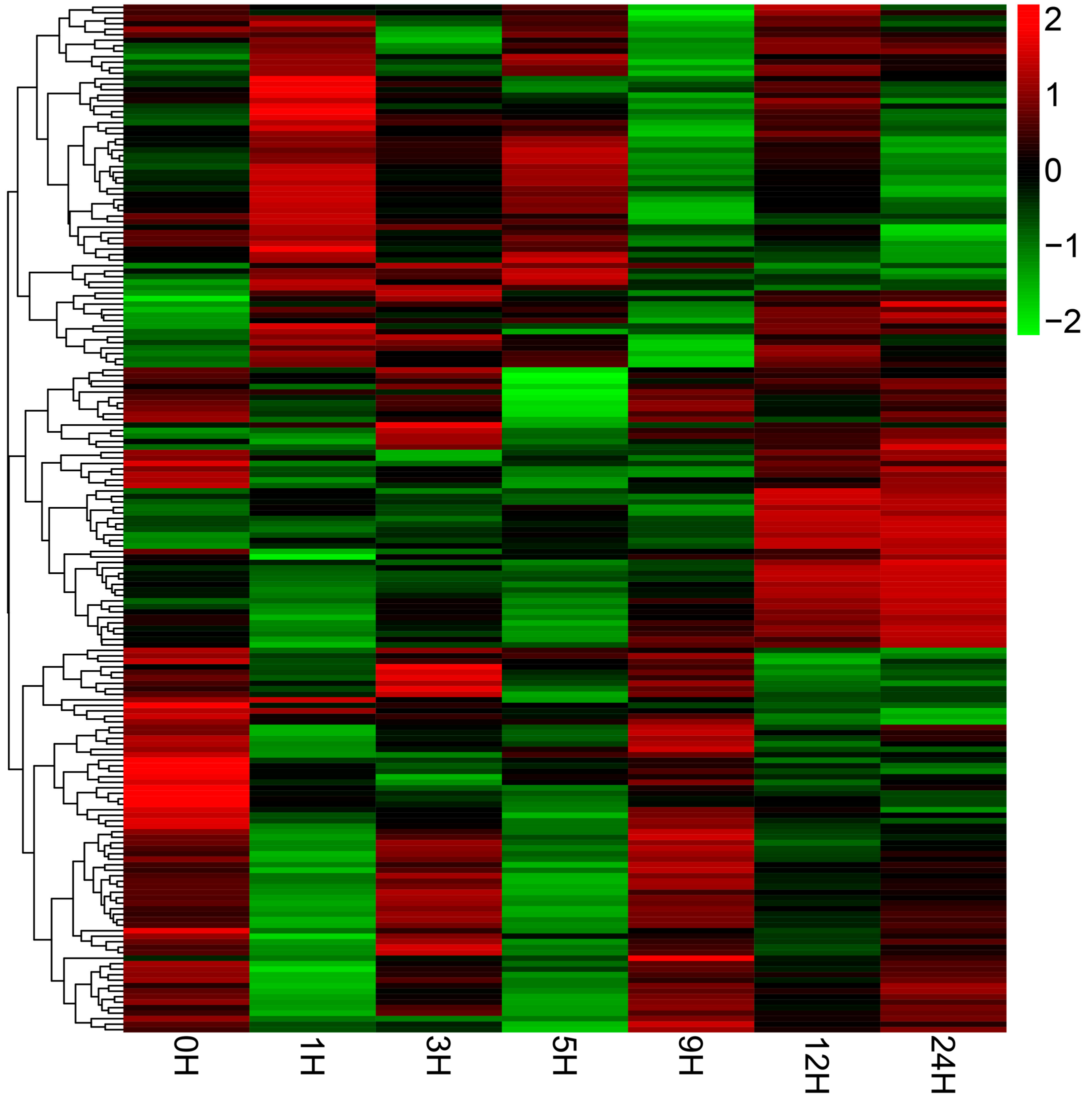

Figure S1. The characteristics of gene expression at different PEG treatment time points in GRN.

The heatmap of gene expression of *P. davidiana×P. bolleana* under drought stress for 0, 1, 3, 5, 9, 12 and 24h.
