# Supplementary material for: Construction of a Hierarchical Gene Regulatory Network to Reveal the Drought Tolerance Mechanism of Shanxin Poplar
Source: Int J Mol Sci. 2022 Dec 26;24(1):384. doi: 10.3390/ijms24010384 (PMC9820611; doi:10.3390/ijms24010384)
Supplement: Supplementary file 1 [file ijms-24-00384-s001.zip › Figure S2 .pdf]

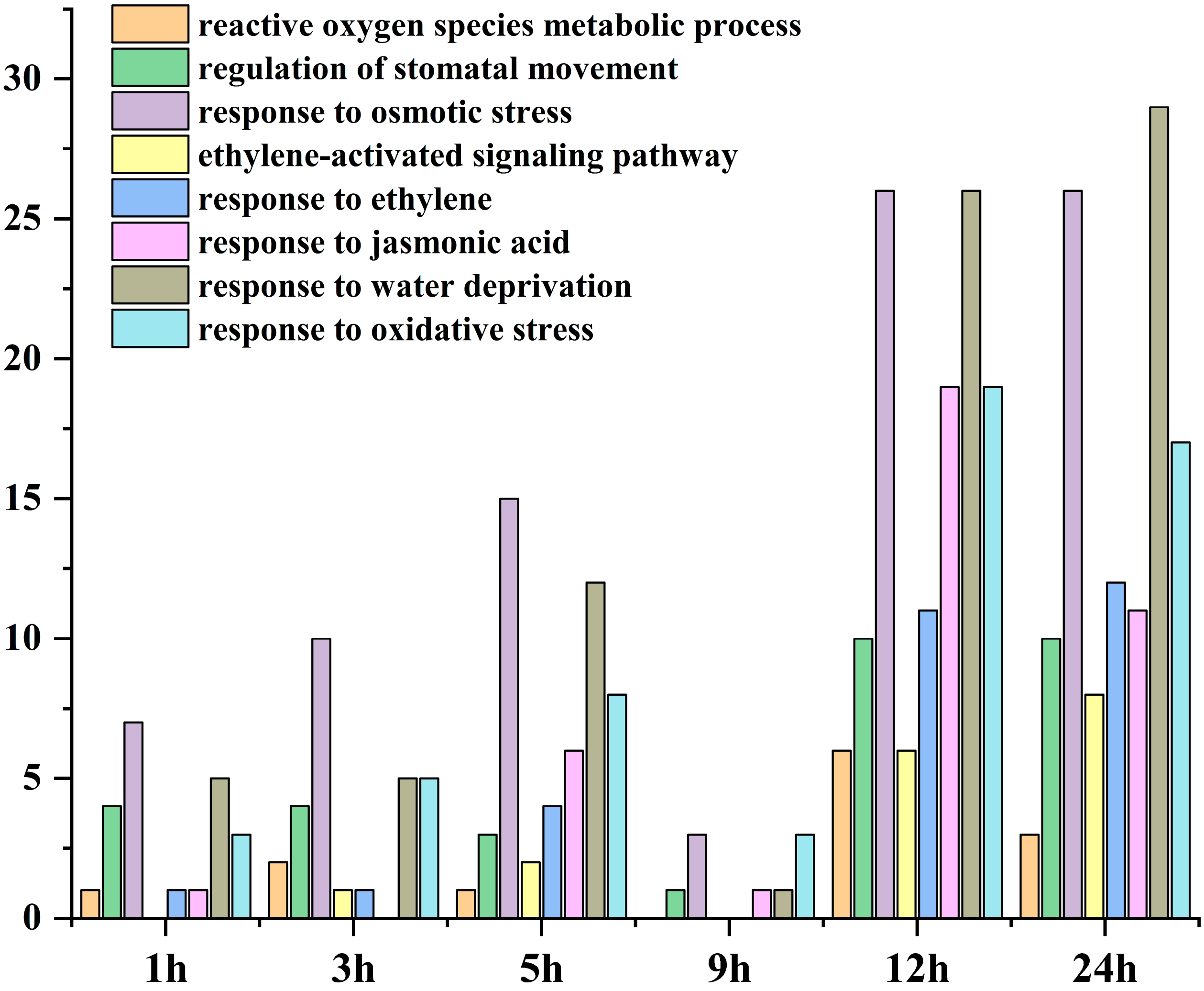

Figure S2. The GO classification of DEGs with different time points.

Biological process analysis was performed.

Every Biological process GO terms in different time point have different gene number in DEG, this figure show that different time point that one GO terms enrichment gene numbers (per gene in GO category see Supplementary Table S4).
