# Supplementary material for: Construction of a Hierarchical Gene Regulatory Network to Reveal the Drought Tolerance Mechanism of Shanxin Poplar
Source: Int J Mol Sci. 2022 Dec 26;24(1):384. doi: 10.3390/ijms24010384 (PMC9820611; doi:10.3390/ijms24010384)
Supplement: Supplementary file 1 [file ijms-24-00384-s001.zip › Figure S4.pdf]

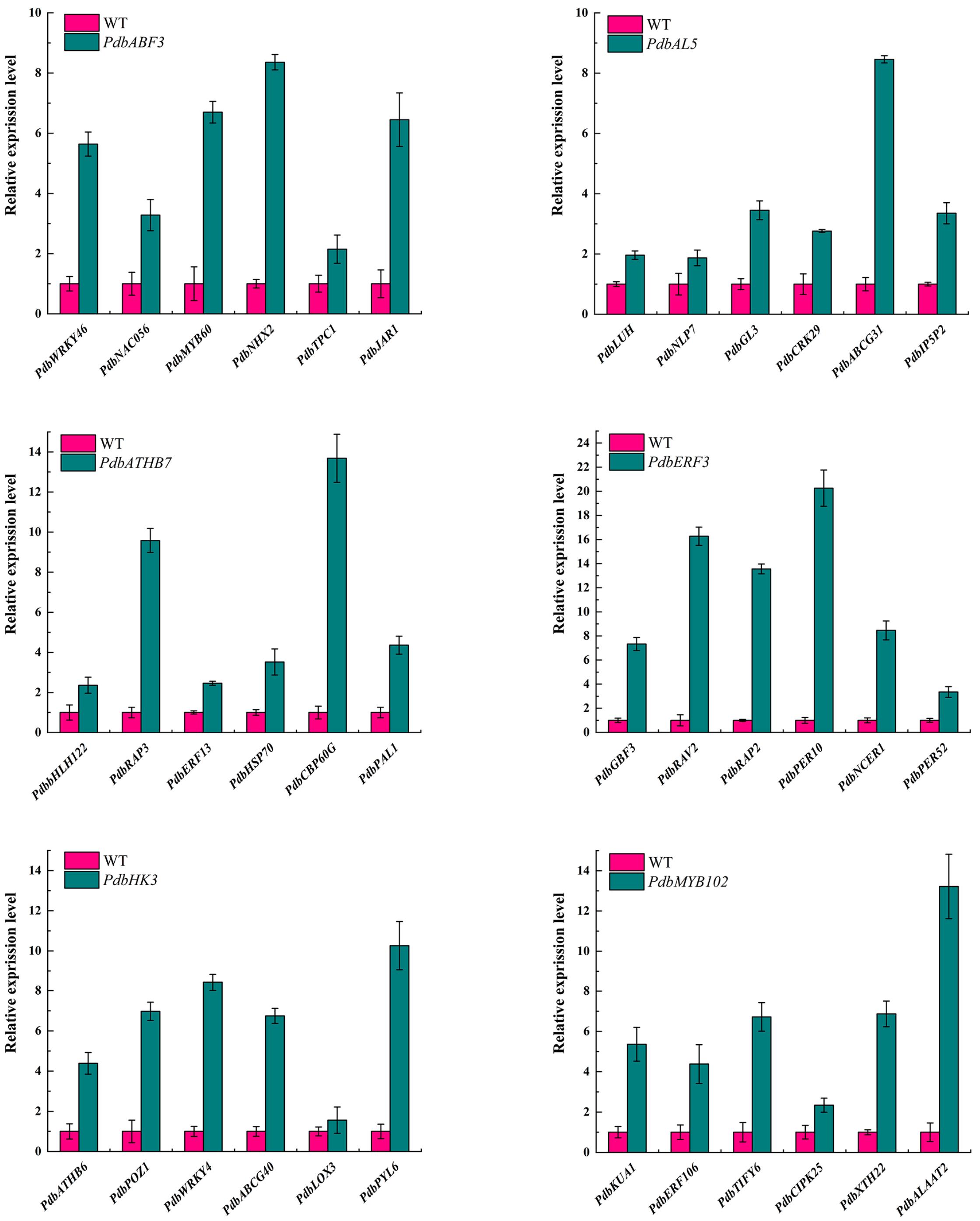

Figure S4. Determination whether the expression of the genes in the second and third layer can be regulated by the 6 TFs in the first layer using qRT-PCR. The genes in the second and third layer were selected for study and gene names were shown as Table S5.
