# Supplementary material for: Construction of a Hierarchical Gene Regulatory Network to Reveal the Drought Tolerance Mechanism of Shanxin Poplar
Source: Int J Mol Sci. 2022 Dec 26;24(1):384. doi: 10.3390/ijms24010384 (PMC9820611; doi:10.3390/ijms24010384)
Supplement: Supplementary file 1 [file ijms-24-00384-s001.zip › Table S2 .pdf]

Table S2. Reads mapping in *Populus davidiana*×*P.bolleana* genome.

Time point	Reads number	Replication 1	Replication 2	Replication 3
Control(0h)	Sequenced	43,865,608	45,294,360	42,643,038
	Mapped	38,919,724 (88.72%)	39,191,930 (86.53%)	37,795,501 (88.63%)
1h	Sequenced	43,199,260	38,491,548	43,842,462
	Mapped	38,352,186 (88.78%)	34,510,399 (89.66%)	39,045,397 (89.06%)
3h	Sequenced	43,963,508	45,008,154	44,373,416
	Mapped	37,679,972 (85.71%)	39,757,770 (88.33%)	38,563,081 (86.91%)
5h	Sequenced	46,273,570	43,906,812	38,940,992
	Mapped	40,953,733 (88.50%)	39,384,310 (89.70%)	35,056,398 (90.02%)
9h	Sequenced	41,359,178	43,576,806	38,812,816
	Mapped	33,908,338 (81.99%)	39,118,035 (89.77%)	34,596,922 (89.14%)
12h	Sequenced	43,250,398	38,402,100	43,229,096
	Mapped	39,096,673 (90.40%)	34,831,191 (90.70%)	39,066,845 (90.37%)
24h	Sequenced	42,490,530	43,246,062	46,491,730
	Mapped	36,311,667 (85.46%)	37,663,831 (87.09%)	40,810,762 (87.78%)
