# Supplementary material for: Construction of a Hierarchical Gene Regulatory Network to Reveal the Drought Tolerance Mechanism of Shanxin Poplar
Source: Int J Mol Sci. 2022 Dec 26;24(1):384. doi: 10.3390/ijms24010384 (PMC9820611; doi:10.3390/ijms24010384)
Supplement: Supplementary file 1 [file ijms-24-00384-s001.zip › Table S7 .pdf]

Table S7. The primers used for vector construction.

Primer names	Primer sequences (5'-3')<br>(Restriction site was underlined)	Restriction site names
35S:PdbERF3-3xFlagF	CTAGTCTAGAATGGCCAGACCGCAACAGCG	*Xba* I
35S:PdbERF3-3xFlagR	CTAGGGATCC(CTTGTCATCGTCGTCCTTGTAATC)$_n$<br>CTGAGTTGCCACCCCAGAAC	*Bam*H I
35S:PdbMYB103-3xFlagF	CTAGTCTAGAATGGGTAGAGCACCTTGTTG	*Xba* I
35S:PdbMYB103-3xFlagR	CTAGGGATCC(CTTGTCATCGTCGTCCTTGTAATC)$_n$<br>CATGAAGTTAATAGCATCCA	*Bam*H I
35S:PdbATHB7-3xFlagF	CTAGTCTAGAATGAATTAAAGATATAGAAG	*Xba* I
35S:PdbATHB7-3xFlagR	CTAGGGATCC(CTTGTCATCGTCGTCCTTGTAATC)$_n$<br>AGCCCAGAAATCCCACCACT	*Bam*H I
35S:PdbHK3-3xFlagF	CTAGCCCATGTCCCGTGTTTGTTACTG	*Sma* I
35S:PdbHK3-3xFlagR	CTAGCCC(CTTGTCATCGTCGTCCTTGTAATC)$_n$<br>ATTCGCACTGGAAGTTGGCTGC	*Sma* I
35S:PdbHK4-3xFlagF	CTAGCCCATGGCTGTGTTGTGTCCCTGTA	*Sma* I
35S:PdbHK4-3xFlagR	CTAGCCC(CTTGTCATCGTCGTCCTTGTAATC)$_n$<br>TGACTTTAAAGTGGATTTGCT	*Sma* I
35S:PdbTIFY6-3xFlagF	CTAGTCTAGAATGGAGAGAGATTTCCTAGG	*Xba* I
35S:PdbTIFY6-3xFlagR	CTAGGGATCC(CTTGTCATCGTCGTCCTTGTAATC)$_n$<br>CTAGTTGCTGGCTGGGAGAG	*Bam*H I
35S:PdbGBF3-3xFlagF	CTAGCCCATGTGAGAGAGTTAAAGCAG	*Sma* I
35S:PdbGBF3-3xFlagR	CTAGCCC(CTTGTCATCGTCGTCCTTGTAATC)$_n$<br>ACCAGCAGCCACAGCATCAGCTCT	*Sma* I
35S:PdbRAV2-3xFlagF	CTAGTCTAGAATGGATGGAAGCTGCATAGA	*Xba* I

Primer names	Primer sequences (5’-3’)<br>(Restriction site was underlined)	Restriction site names
35S:PdbRAV2-3xFlagR	CTAGGGATCC(CTTGTCATCGTCGTCCTTGTAATC)$_n$ CACCTCCTGACCCCCCCTCC	*Bam*H I
35S:PdbWRKY4-3xFlagF	CTAGCCCATGACCAAAAATGGAAAGGA	*Sma* I
35S:PdbWRKY4-3xFlagR	CTAGCCC (CTTGTCATCGTCGTCCTTGTAATC)$_n$ TGTTATCTGCTCTTCTTTTAAC	*Sma* I
35S:PdbKUA1-3xFlagF	CTAGTCTAGAATGACGAGGCGGTGCTCGCA	*Xba* I
35S:PdbKUA1-3xFlagR	CTAGGGATCC(CTTGTCATCGTCGTCCTTGTAATC)$_n$ TGGATTGGGCTGCTGCTTCT	*Bam*H I
35S:PdbNAC056-3xFlagF	CTAGTCTAGA ATGAACCACCTCTCTAAATT	*Xba* I
35S:PdbNAC056-3xFlagR	CTAGGGATCC(CTTGTCATCGTCGTCCTTGTAATC)$_n$ AGAGTACCAGTTCATGCCTG	*Bam*H I
35S:PdbGL3-3xFlagF	CTAGTCTAGA ATGGAAAATGAGCTCCGTAA	*Xba* I
35S:PdbGL3-3xFlagR	CTAGGGATCC(CTTGTCATCGTCGTCCTTGTAATC)$_n$ TCGATTCGAGAACAAGTTCCCA	*Bam*H I
35S:PdbMYB60-3xFlagF	CTAGCCC ATGGGAAGACCTCCTTGCTG	*Sma* I
35S:PdbMYB60-3xFlagR	CTAGCCC (CTTGTCATCGTCGTCCTTGTAATC)$_n$ GAATATTGGAGACAGTTCCA	*Sma* I
35S: PdbLUH-3xFlagF	CTAGGAATTC ATGGCGCAGCAGAGTAATTG	*Eco*R I
35S: PdbLUH-3xFlagR	CTAGGAATTC(CTTGTCATCGTCGTCCTTGTAATC)$_n$ TCACCATACGGCCACATTCT	*Eco*R I
