# Supplementary material for: Construction of a Hierarchical Gene Regulatory Network to Reveal the Drought Tolerance Mechanism of Shanxin Poplar
Source: Int J Mol Sci. 2022 Dec 26;24(1):384. doi: 10.3390/ijms24010384 (PMC9820611; doi:10.3390/ijms24010384)
Supplement: Supplementary file 1 [file ijms-24-00384-s001.zip › Table S8 .pdf]

Table S8. The primers used for ChIP-PCR.

Primer names	Primer sequences (5'-3')
Pda_00008321_1F	TAATTTTCAATTCTCATTTACAAC
Pda_00008321_1R	GCAAATGTCTAGAAAGAAAG
Pda_00008321_2F	CCCCAGATTTTTCATCTTTCTTTAC
Pda_00008321_2R	ATTCAAAGCTATTTTGTTTA
Pda_00008321_3F	CAATATTTATAAAATATGTTTT
Pda_00008321_3R	AAAAAGTTCTTGAAAATTTT
Pda_00008321_4F	TTTGAATGTAAAATAAACACA
Pda_00008321_4R	CTTTCTCCAATTTCAACACG
Pda_00020467_1F	ACAATCACAAAATCGAGTGTCA
Pda_00020467_1R	TCTTGAATTTGTCCTCTGGA
Pda_00020467_2F	ACAACCCAACTACCCGTCAAT
Pda_00020467_2R	TCTTTTGCATCCGAAATTTA
Pda_00020467_3F	AGACAATCTTCTTCTCATCAT
Pda_00020467_3R	AGCCTTATCAAAATAAACCC
Pda_00020467_4F	AATATAATAGAATATTAGCAGC
Pda_00020467_4R	TATCTTAAGCTCAAAAGGAT
Pda_00034750_1F	GCATCTCGATATCCAAAACAT
Pda_00034750_1R	GAATCTGAAGCACCACCCTA
Pda_00034750_2F	GTCCATGAACGCTTTAAGCCC
Pda_00034750_2R	TGGGAACTTAGCACAATAAT
Pda_00034750_3F	GAAAACTCAAGCTCAACATTGT
Pda_00034750_3R	TACAAATTGCCTTAATAAAA
Pda_00034750_4F	TATAAATAATGATTTGTATATT
Pda_00034750_4R	ATGGCTTTGTTATTAGAGTA
Pda_00019195_1F	TCTGCAGTCTGCCCTCCTTTAG
Pda_00019195_1R	TCCTTGAGTGCTTTTTATTC
Pda_00019195_2F	AATATGTTGGACTGGTTTACGT
Pda_00019195_2R	ATCATGCCATTTGAATTACC
Pda_00019195_3F	CAAGCAACAAATGTAACTGAT
Pda_00019195_3R	GGACATAACTTCTCTTCTTT
Pda_00019195_4F	ACGGTAGAAACAAAAACCTA
Pda_00019195_4R	CTGCAAACAAAAACTTTGAT
Pda_00019597_1F	TTTTTCACACCTCAGGGCAAGACC
Pda_00019597_1R	GAGAAAAGAATTTTTTTAAG
Pda_00019597_2F	ATGAGAGCTCAAAATTAACCA
Pda_00019597_2R	ATTACTACTCTGTGATGTCT
Pda_00019597_3F	TCTACTGCATTGCTTCACGTAC
Pda_00019597_3R	GTGATTCATTAAATGGATTT
Pda_00019597_4F	AAACCAGTCCCCAATCATTTAT
Pda_00019597_4R	TTGATCTTAGTCTTGTTTTC

**Primer names**	**Primer sequences (5’-3’)**
Pda_00037495_1F	TCCACACTACCTATACTTTATCAA
Pda_00037495_1R	TTATCCTTAACAGGTGACAC
Pda_00037495_2F	TATATCCTAGTACTTAAAA
Pda_00037495_2R	AAATTAAGTGAATATTACAA
Pda_00037495_3F	TGATGTTAAAATAATATTTAA
Pda_00037495_3R	TTTCAAAAAATTTCACAAGA
Pda_00037495_4F	GATTTTAATTTAACATTATAGT
Pda_00037495_4R	TCACTGAGCCAATCAGGAGC
Pda_00034144_1F	ATACTAGACAGGATAATAATA
Pda_00034144_1R	CTTGATCTAAAAGATTAAAA
Pda_00034144_2F	TAACAACCAGCTTTCAACTTT
Pda_00034144_2R	GAAATTCAGCGCACCCTTCA
Pda_00034144_3F	AAGGGCAGGAGTCTTGAATC
Pda_00034144_3R	TTTCTTGATCCCTCCCTATC
Pda_00034144_4F	CACACCACAAAGGAAGACCA
Pda_00034144_4R	TTCCTGATAATTAGTACTTG
Pda_00023176_1F	GCATTACCAGAAAGATAGT
Pda_00023176_1R	TAGCCTCGGGTTGGGGAACA
Pda_00023176_2F	ACTCCAAACCTATAAAAATAA
Pda_00023176_2R	CCAATTAACTTGAATTTGTC
Pda_00023176_3F	CTAATTAGTTTTGAAACTTTTT
Pda_00023176_3R	TGTATATATTATGTCCTTAT
Pda_00023176_4F	ATTAAAAATATAGGGATATA
Pda_00023176_4R	TGTCTCTTCAATGAAACAAC
Pda_00034049_1F	ACACGTGAATTGAAAACTATG
Pda_00034049_1R	GAAAAATTAAGGCATCACAT
Pda_00034049_2F	TATAGTTTAAGTAACATGGAT
Pda_00034049_2R	TATTTCAAGTCTAAAAGGTC
Pda_00034049_3F	CTAATCTAATATAGAATAATA
Pda_00034049_3R	CAAATCAGGTTTGTAAGATG
Pda_00034049_4F	CCGCGAAGTCCGTAGCGACAT
Pda_00034049_4R	TGGCTAAGGACCTCCACCCA
Pda_00037432_1F	AACCACCATACCCCCATCGT
Pda_00037432_1R	GATTTTAAGAGAAAGAGGAGAGA
Pda_00037432_2F	CCGTGGTTTTTTGAAGATTTCTTTTT
Pda_00037432_2R	AAAGCTTCAGTTATAAACTA
Pda_00037432_3F	CCTGATAAAATTATTAGCCAGAA
Pda_00037432_3R	AATTTACAGGTAAAACTTT
Pda_00037432_4F	ACACAGTTTCTTAAACAGTGTT
Pda_00037432_4R	CTGCAACAAAAGGAGACC

Primer names	Primer sequences (5'-3')
Pda_00010869_1F	CAACATATTTATTTATTTGTT
Pda_00010869_1R	CGTAAAAAAAACACTAAAAA
Pda_00010869_2F	ATTTCCTTATCTATGATTTTTTC
Pda_00010869_2R	ATAAAATTGAGATAATTTCT
Pda_00010869_3F	ATTTATTGTTGTAGAGTTTAAT
Pda_00010869_3R	TGAACATCTCATGCAATTAC
Pda_00010869_4F	TACCCCCAATTTAATAATTTAA
Pda_00010869_4R	CTTTGCTTCTGGTTCAGGTT
Pda_00025105_1F	CCATTCATGTTTCCCATCGA
Pda_00025105_1R	AGGTTGGAAACACAATTGGT
Pda_00025105_2F	ATTATTAATTATTATTATTACGA
Pda_00025105_2R	ATATTGCATAAAAATGAACT
Pda_00025105_3F	ATACTCGCTGCAGTGTATCAA
Pda_00025105_3R	TGCCCGTCCTTGAAGTGCAG
Pda_00025105_4F	TGTAAGCCAACAAAAACATCAC
Pda_00025105_4R	TCCTAGCAAAAGCAAGAGCC
Pda_00025305_1F	CCATTCATGTTTCCCATCGAG
Pda_00023176_1R	AGGTTGGAAACACAATTGGT
Pda_00025305_2F	ATTATTAATTATTATTATTACG
Pda_00025305_2R	ATATTGCATAAAAATGAACT
Pda_00025305_3F	ATACTCGCTGCAGTGTATCAA
Pda_00025305_3R	CTGCACTTCAAGGACGGGCA
Pda_00025305_4F	TGTAAGCCAACAAAAACATCAC
Pda_00025305_4R	TCCTAGCAAAAGCAAGAGCC
Pda_00000550_1F	AGTTGCCAATTGTACCAGAT
Pda_00000550_1R	ATCAATATAATTTTTTATAT
Pda_00000550_2F	GTATTTTTAAAAAAAAAATTAA
Pda_00000550_2R	TTAATTATTGAGTTTTGATC
Pda_00000550_3F	TTAATTAATATTTTATTTACAA
Pda_00000550_3R	ACCAGCTTATGTCATCCATT
Pda_00000550_4F	GAAATCACACCCCCACATTATCA
Pda_00000550_4R	TTCTTCTTCTTCTTCTTCTC

Primer names	Primer sequences (5'-3')
Pda_00011148_1F	ATTGTCTTAAAATTCAAGGG
Pda_00011148_1R	TCTTATAAATGTTCTAATTA
Pda_00011148_2F	ATTTACACTTCAAAACATAC
Pda_00011148_2R	AATTAAATGTTAGAAAAAAA
Pda_00011148_3F	TTTTTCTTGGAACAATCTCC
Pda_00011148_3R	AAGACTGCGTCTTTGATCAA
Pda_00011148_4F	TTTTCTTTTCTTGTTTTAAT
Pda_00011148_4R	TGTTTGCTGAAAGGGACAGA
Pda_00028012_1F	ATAAAAAAAACATCTAAAAGT
Pda_00028012_1R	ATTTTTTTTATTTCATCATT
Pda_00028012_2F	TACAAAAGGAGCAAGAAAAA
Pda_00028012_2R	ATAGTGTAATTAATAATCTA
Pda_00028012_3F	GCTTAAGAAAATAAAAAAACTTA
Pda_00028012_3R	TTTGTTTTAACAAGTTTTAA
Pda_00028012_4F	AATCCAAAATAAAAATATTG
Pda_00028012_4R	GTTAAAATTCTTACAGCACT
Pda_00019367_1F	CGACGAGTTCAACTTTCAGG
Pda_00019367_1R	CTTTAATCAATTTTTTTTTA
Pda_00019367_2F	GTTTTATGTCATTTTTTATA
Pda_00019367_2R	CACTTCAGTGCTTCCCCTTT
Pda_00019367_3F	TCCAAATTGGTACAGAAACAA
Pda_00019367_3R	CAATAAGACTTCTCTTTATT
Pda_00019367_4F	ACCTGCAGACACTACTCTTG
Pda_00019367_4R	ACAATTATCTCATGGAATAA
Pda_00006818_1F	TCAGAAGGTCTCGGCTCATTT
Pda_00006818_1R	AATGGCATCTCCTACTTTTA
Pda_00006818_2F	TTTGTCATGGATCCATTACT
Pda_00006818_2R	CCGACGTGGAGAGACCATCC
Pda_00006818_3F	ATACCCCATTGTAATACTTA
Pda_00006818_3R	CACTCTCCACTGGTCGCCTC
Pda_00006818_4F	AGGTAATAAATTCATTTGAG
Pda_00006818_4R	TTATTGATCCTCAAAAATCC
Pda_00031022_1F	AAAGAATAAAATTCTAGATT
Pda_00031022_1R	TTTTAACTAATACCCCATTA
Pda_00031022_2F	ATTAAATTCAAAAAATAAATG
Pda_00031022_2R	CGGTCGACCATGTTATGGCT
Pda_00031022_3F	GACACTTTCTTTCTCCTCTT
Pda_00031022_3R	ATCGTCTCTTATCATGTAAA
Pda_00031022_4F	ATCCCATGTAAAATAGGGTT
Pda_00031022_4R	TTCTCGAAAAACTCCTACTG

Primer names	Primer sequences (5'-3')
Pda_00020570_1F	ATTTTTATTATTATATGATT
Pda_00020570_1R	TATATTTGAGTCCAAATTAA
Pda_00020570_2F	CCTATTAAATATCTTTTCAA
Pda_00020570_2R	GCTAATATGATGATCATTAG
Pda_00020570_3F	AATCATTAGACTCAAACTTT
Pda_00020570_3R	CCTACCGTATTAAAAAGATG
Pda_00020570_4F	ACATGGTTGTCAATTTCAGT
Pda_00020570_4R	TCCTGCCTCTCTCCCTCTTT
Pda_00036271_1F	GTGTTCCTCTCACCCCTGTG
Pda_00036271_1R	CCTATTACACAACTTCTTTT
Pda_00036271_2F	AAACTTGAGATTTAATTAAA
Pda_00036271_2R	GAATCTTATTATTTTTATTT
Pda_00036271_3F	AAACTCATGATCGCTTGCGCTT
Pda_00036271_3R	CTCAAAAATATTATAAACAA
Pda_00036271_4F	TGGGGAAAAAGAATAAAATA
Pda_00036271_4R	TGAAAGAACTAAAAATTTCG
Pda_00000970_1F	TAAATAACTTGTATATTAAC
Pda_00000970_1R	TCTTTTTTCTCTAAGAGATT
Pda_00000970_2F	GACACTGGTACTTGTTGAAT
Pda_00000970_2R	GCTTTTACAGGGGGAGGCCG
Pda_00000970_3F	CATATTCTTAGGGTTCTTGG
Pda_00000970_3R	CCATTAATTGTGAGCGCAGG
Pda_00000970_4F	ACAATATTTTATACTACGCAAAAA
Pda_00000970_4R	TTTCTGCTAAGTGTGATTAT
Pda_00002376_1F	GTTTAGAGTATGCCATCATGT
Pda_00002376_1R	CTCGAATTGAAATAATAAGA
Pda_00002376_2F	AACTCACTTTGTTATCACCTTC
Pda_00002376_2R	ATAATTAACCAGAGAAAAAT
Pda_00002376_3F	CTCCTTTGTTTTATTATAGTA
Pda_00002376_3R	ATTTTACATGTTTGATCATT
Pda_00002376_4F	CAAACAAAGCTTAAAATAGG
Pda_00002376_4R	TTCATTTGTGGGATTTTGAT
Pda_00003683_1F	ACAAACCGAACTTAATTTTA
Pda_00003683_1R	ATTTATCAGTTCAAATCATG
Pda_00003683_2F	TAGTGGATATATATATAAAC
Pda_00003683_2R	TTTAATGTCTGCTACAATGT
Pda_00003683_3F	TTCATCCATCTGATCCATTT
Pda_00003683_3R	TTTTTTTGTTTTACGGTGTC
Pda_00003683_4F	ATTAAAAAAATAAAAAGAAA
Pda_00003683_4R	TTATTTCTGTCCGTGGGAGA

Primer names	Primer sequences (5'-3')
Pda_00025510_1F	ATTATATCCGCAAACAATTT
Pda_00025510_1R	TCGACTAATCTCATGGGCCT
Pda_00025510_2F	AATACGTACAAACTGACCAT
Pda_00025510_2R	TAGAATTATATAAAATTAGC
Pda_00025510_3F	AATTTAAAGATAAAATCACA
Pda_00025510_3R	GAGAAGGATTGAAGCAGGTA
Pda_00025510_4F	TGTTTCCCTGGAGCTTTCTATT
Pda_00025510_4R	TTTAACCTGGAAACTTGGAT
Pda_00005883_1F	TCCAAGGTATGCGTTTGAGA
Pda_00005883_1R	CCGCAAGGAGTTTTTTAATT
Pda_00005883_2F	TACAATATGGTCATTTGATT
Pda_00005883_2R	TGCAAAACATGTAATAAATC
Pda_00005883_3F	TCTTACAAGATCTTGATTTAAT
Pda_00005883_3R	GCCCTTAGAAAAAAGAAAAT
Pda_00005883_4F	TCGTTCCAAAGTTTATTTAAG
Pda_00005883_4R	GAAAACAAACCCAAGAAAAA
Pda_00025937_1F	TCTTTATTCTTTTCTTCATTT
Pda_00025937_1R	ATTTAGAAAAATTTAGATTA
Pda_00025937_2F	TATTTATCTATAACAATTAGAAA
Pda_00025937_2R	AGGTAATATTGGGCTGAGCC
Pda_00025937_3F	TTTACATTGCTATTTACCAGCA
Pda_00025937_3R	TATACTTCATTTTGTGATAT
Pda_00025937_4F	ATTGTTTAACAATCGGACTAA
Pda_00025937_4R	TGTTTAGATGGCAGTGATTT
Pda_00006007_1F	GTTACAACAAGCATCACCAG
Pda_00006007_1R	AATCCCACAAGTTTCTGAGT
Pda_00006007_2F	TCACAATTTTTTATCATATTGG
Pda_00006007_2R	AGAAAGGAAAAATGGATGTT
Pda_00006007_3F	ATGCCAACATCCTAATAA
Pda_00006007_3R	AGTTATATTAAATATTAATT
Pda_00006007_4F	CTAGAATAACCTTTGAAATATC
Pda_00006007_4R	TCCAAAGTCAGCAACTTTTA
Pda_00022729_1F	GACCACACGAGCGGTCCA
Pda_00022729_1R	CTCATGTTTTTCTTTACTTA
Pda_00022729_2F	CCATGTAAAAATTTATGGTCAA
Pda_00022729_2R	AAATGACACTTGCCAAATCA
Pda_00022729_3F	GATCAAAGGGCCCCATCGTG
Pda_00022729_3R	TCCACCTAAGCTTAATGAAT
Pda_00022729_4F	ATGGAGTTTGGGATCAAATTAA
Pda_00022729_4R	TGTTTCCTATATTTGTCTCA

Primer names	Primer sequences (5’-3’)
Pda_00040666_1F	CAAGGAAAGTCCCTTTTCCC
Pda_00040666_1R	ATAAGATAGAAACTTAGTTT
Pda_00040666_2F	AAATAGATTGCATGAATCAT
Pda_00040666_2R	TGGTTTAATATTGTTTTTCT
Pda_00040666_3F	TGCTTGATAAATCTTTTGAA
Pda_00040666_3R	TTGCAAAGAACAGAACTTTA
Pda_00040666_4F	GAAAGTTGTGTTTTTTCTTTC
Pda_00040666_4R	TATTCAATTATCAAAACTTA
Pda_00032092_1F	TGGTTTCCAAATTCTATTGA
Pda_00032092_1R	CTATTTTTTTCAAAAAAATA
Pda_00032092_2F	CCAGATTAACCCTTTCAATCTACA
Pda_00032092_2R	TTATTTTAATATATTTTTAA
Pda_00032092_3F	TATATTTTTAAAAAATACTAA
Pda_00032092_3R	TGTTTCACCCTTTGTTTATC
Pda_00032092_4F	TGAGGGAGAGTTCAATTTGTAGTT
Pda_00032092_4R	AAGAAGTCTGCAAATCAGAA
Pda_00016968_1F	ATTTTTTTCGCGAGAATATG
Pda_00016968_1R	TCCATTAAAGGATGCGGTAT
Pda_00016968_2F	GAGTACTCTAGCCAAGGAAA
Pda_00016968_2R	CTCAAATCTACTAGTAGTCT
Pda_00016968_3F	GAACTTATCTTTGAAATAAC
Pda_00016968_3R	TTTCTTCTCAATAATATATA
Pda_00016968_4F	AAAGTTAAAACCTCAAGTTAC
Pda_00016968_4R	TGAAGTAATTACTACTCGCC
Pda_00008662_1F	TTTCAGGGTTTATAAAATTAG
Pda_00008662_1R	TTGTTTTAATCTCTATTTCC
Pda_00008662_2F	GCTTAAAGTAGAAAAAAAAT
Pda_00008662_2R	TTTGATTTTTTGTATCAAGC
Pda_00008662_3F	CCGAGTTCAAATTTATTTATT
Pda_00008662_3R	ATATAAAAGTAAAAACTCCG
Pda_00008662_4F	ATATATAGCATGAGAAGTCTAT
Pda_00008662_4R	TGAAAGTTGGAGATCAAGGT
Pda_00036802_1F	CTATTCCTGATGAAACTGAACA
Pda_00036802_1R	AATTATTTACATATTTGCTA
Pda_00036802_2F	CAATTTTTTTGTCGTGTATATA
Pda_00036802_2R	TTAATTTTGTTAAATTTGAT
Pda_00036802_3F	ATATTTGTTTTATCAAAAAATAG
Pda_00036802_3R	CACGTATTTTTTTGTTTTGG
Pda_00036802_4F	GGCCTCTTGGTTTTTGTTTGAA
Pda_00036802_4R	CCTAAAACGTTTGAAATGGA

Primer names	Primer sequences (5’-3’)
Pda_00037420_1F	TCTAATATATATCCTTATGCCA
Pda_00037420_1R	TTTCTGAAGTAAAAACATTG
Pda_00037420_2F	TTAGAATCTTGTCCGTGGGTT
Pda_00037420_2R	GAAAAGGAAACAAGTGGAAG
Pda_00037420_3F	CTTGATAATAACCAACTCTTT
Pda_00037420_3R	AGAAAAGAGAGTCGAAATCT
Pda_00037420_4F	ATATCTCTTCTTAGTCCATATC
Pda_00037420_4R	CTGCAATCAGAACATCAAAA
Pda_00003956_1F	CTAGATGTTTAACATGTCAT
Pda_00003956_1R	ATGGGTTTCTGATTTCTTGT
Pda_00003956_2F	GTTGTTTGTGTTCAATATTCC
Pda_00003956_2R	TTATGGATTATCGACAGAAC
Pda_00003956_3F	ATTTTTGCAAAACAAGTCCAT
Pda_00003956_3R	ACGGTTGCAGTAGAAAGGAA
Pda_00003956_4F	CAGAACAACGCAGCTGATGT
Pda_00003956_4R	TTTGATCACGAGTTTATTCT
Pda_00007115_1F	TAATTATCTGTCAAAGTCTA
Pda_00007115_1R	GATTAGTTGAAATGTATGTA
Pda_00007115_2F	CCATTAGGTCTGAAGTTAAC
Pda_00007115_2R	TGTCGAGGAAGATGAGATGG
Pda_00007115_3F	ACAAGAACAACACGGACAACATAT
Pda_00007115_3R	CATATTGTTATGATTATTAT
Pda_00007115_4F	AATTTGCATGCTAAGAGTTAAT
Pda_00007115_4R	TATCGGTTTCAACTTTCAAG
Pda_00040672_1F	CCAGTCTTTTATATCAAATTAA
Pda_00040672_1R	GAATAACTAAAAAGACTTTT
Pda_00040672_2F	TAAGCTTAAATGTGCAATAGA
Pda_00040672_2R	AAAATCGTAGTTAATCAGAA
Pda_00040672_3F	CTCAAGTGTGGCTGCTTGAGGAGG
Pda_00040672_3R	AACCAATTATTCTTTGATTT
Pda_00040672_4F	GGTATTTGGGATACATTTTAC
Pda_00040672_4R	CATAAAAAGTTGCCCTGAAA
Pda_00002023_1F	AAAATACCAAATTTTGGCGA
Pda_00002023_1R	ACCACAGCCTTACAGTTGAA
Pda_00002023_2F	GTTTATATTTTATAGCTTTGTTA
Pda_00002023_2R	ATGTTAGCCTAAAACCCATG
Pda_00002023_3F	AGCCCAGTAGCTATTGATAGA
Pda_00002023_3R	AACATGTTAAAAATACTAAA
Pda_00002023_4F	AATATCAATGAATATTTATGG
Pda_00002023_4R	TCGGTAAGAAAGATTTTTCG

Primer names	Primer sequences (5’-3’)
Pda_00005846_1F	TAGGGCTCACGCGCAGAGAG
Pda_00005846_1R	TCATTCAGGAAAATTCATGT
Pda_00005846_2F	ATCCAAAAACAATTAAAAAAA
Pda_00005846_2R	AACCAGGTTTTTCTGAAGTT
Pda_00005846_3F	TTTATTTATCCTGATTTTTTA
Pda_00005846_3R	CCATTCAAATTTACTACAAT
Pda_00005846_4F	CTTTTTAACTGTTCAAGTAA
Pda_00005846_4R	TGTTGATGCTAGGAAATATG
Pda_00037217_1F	AGTGAGAGTACGAGTAAAGA
Pda_00037217_1R	TAATTAGAGAATCAACTGAA
Pda_00037217_2F	TGAAAGAAATACTACAGTTTCT
Pda_00037217_2R	AGGTTGTCTTTTCGATATCT
Pda_00037217_3F	ATTTAATAGTTGTAATAGATTAC
Pda_00037217_3R	AGAAGCCGCCACGTGGGTGG
Pda_00037217_4F	GGCTGTGTACCCTCCTTCATG
Pda_00037217_4R	TAATTTGATGATCAACAAAC
Pda_00026826_1F	GTGACGTGGGAAATGTTTTTT
Pda_00026826_1R	TTTTAAAATAAATTATAAAT
Pda_00026826_2F	AACAAAATCATGACCGGAAG
Pda_00026826_2R	ATTGTACATACATCGATTAA
Pda_00026826_3F	TTAGCACTAACACCCACATTA
Pda_00026826_3R	AAGGATGATTATGGCCTTTC
Pda_00026826_4F	GCCCACACACGAGGCCAAGT
Pda_00026826_4R	TCTCTAACTTATAAATGTAA
Pda_00011485_1F	AGTGTGTTTTGTTGAGAGGG
Pda_00011485_1R	AATTGAGTTCATCCAGAATG
Pda_00011485_2F	GTCTTTTGATTTCCATTCTTTGGT
Pda_00011485_2R	GCCAGTGACGTGCACAGAGA
Pda_00011485_3F	ACTTATCCATGAGCCATGGCC
Pda_00011485_3R	AGAGAGGCTCAAAACTCAAA
Pda_00011485_4F	TTCCATTCTTTTGTTGTGCTCGT
Pda_00011485_4R	AGAGTCAAGAACCAAATAAC
Pda_00028118_1F	TAATCTTTATTTTTTTATTTGA
Pda_00028118_1R	GATTAAATTTTCTTTTAAAA
Pda_00028118_2F	TTTTTTCAGTCATGTCCTTCAAA
Pda_00028118_2R	ACGAACCAACCGATACGTGG
Pda_00028118_3F	CTCGGTTTTAATTAAAAATAA
Pda_00028118_3R	TGGCATTTATATAGGGAGGA
Pda_00028118_4F	AAATCACATCGATTAATCATC
Pda_00028118_4R	ACTGCAAATTCAAAACAGCG

Primer names	Primer sequences (5’-3’)
Pda_00009232_1F	GAGCTAATTAAGAATTCAGTT
Pda_00009232_1R	ATAAAATATTATTAAAAAAA
Pda_00009232_2F	TTTTTTAATATTAAATTGATTAA
Pda_00009232_2R	CTTTCCTGATTTCATTTAAT
Pda_00009232_3F	ATAAAAAATTAAATAAATCAA
Pda_00009232_3R	GAACACCCTAAATGCATATC
Pda_00009232_4F	ATCTGATCATAATTGACAATGG
Pda_00009232_4R	ATTGAATGCTTTGGAACTTC
Pda_00010167_1F	GATTTTGGAGTAAGTGTAACTT
Pda_00010167_1R	AAACATTTAAGGGATTGTTT
Pda_00010167_2F	CATTATTCTAAAATCAGGTCC
Pda_00010167_2R	TTTATTTTAAAAAAATTATG
Pda_00010167_3F	ATTTTTAAATAATATATTAAA
Pda_00010167_3R	TACATGATGGCTGGTTAAAA
Pda_00010167_4F	CACGCGTCCAGCCTTCTCCCC
Pda_00010167_4R	TAGCAGAGGAAAACAGAGCA
Pda_00022832_1F	ATAACAAAAGAAAAAAAAAG
Pda_00022832_1R	AAAAGAAATCATAAGCGTCC
Pda_00022832_2F	TTCTTGATGGAGAAAAAAGGA
Pda_00022832_2R	TACATTCCTTATTTAAAATG
Pda_00022832_3F	GGCTTTATTTCAGAGAATGAAAA
Pda_00022832_3R	TCCTATCCAGATTTGTTTTT
Pda_00022832_4F	ATATTTTTATTATTTTTTTGAATA
Pda_00022832_4R	TCTTTTAACTCTAGCCGAGA
Pda_00012337_1F	CATACTCCTCTAAGTTTCCAT
Pda_00012337_1R	ATATAAAAATAAATCTAGAT
Pda_00012337_2F	GAAATTAATTTTTTATAGATTA
Pda_00012337_2R	TAAAACTAAGAATATGTTAG
Pda_00012337_3F	GCGCAAAATAGAAATGAATTT
Pda_00012337_3R	TGCATGGATATAAAAATGAA
Pda_00012337_4F	ATGAGGAATTGCTTCAAAAACC
Pda_00012337_4R	TGACTGAAACAACTGAAAGA
Pda_00019030_1F	ATTAAGCAGTGATTTCATTAAC
Pda_00019030_1R	AAAAATAAAATAAAAAATAT
Pda_00019030_2F	AAAATATATCAAAATAATTTAA
Pda_00019030_2R	AATTAGTGCTCCTGTGCAAG
Pda_00019030_3F	TAGGATTATTGAAAAATAAAGA
Pda_00019030_3R	TCAATTTTGAAAATAAAATA
Pda_00019030_4F	GAGTTTTTTTTTTTGGTGGTGAA
Pda_00019030_4R	CGTGAACAAACGCTGGTAAT

Primer names	Primer sequences (5'-3')
Pda_00010739_1F	ACAATTTACACCACGTAGCACAC
Pda_00010739_1R	TAAGGACATATTGTTAAGAA
Pda_00010739_2F	TGATTTCACGATATTCAAAGA
Pda_00010739_2R	CTTTATTAATTGTCTCATAT
Pda_00010739_3F	TAAATTTTTTAATTTTTAATTTTTA
Pda_00010739_3R	CGTCATCAGTCCCGAGCACG
Pda_00010739_4F	TTGTCCAAACAAAACCGTCAGAT
Pda_00010739_4R	CGAAAGAAAGATTTTTCTAA
Pda_00005860_1F	TTAATTATCAATATTATGCATTT
Pda_00005860_1R	CAGAAATGCCCTCGTTGAGA
Pda_00005860_2F	TACTTCAAAAACTCCACTACCC
Pda_00005860_2R	CTTCTTCTAATTTTTCGCAA
Pda_00005860_3F	AAAAGAGATAGTTCGAAAAAAG
Pda_00005860_3R	CTAAAAGAATTATCAATTCA
Pda_00005860_4F	TTTCTCGACATTGACTGCATTTTT
Pda_00005860_4R	TTTCACCAATCAGGGAACCT
Pda_00005807_1F	TTCTCTTCATTTAAAAAGTGTGG
Pda_00005807_1R	ATATATTAAAAAAAACACTT
Pda_00005807_2F	CAAAATAATATTTTTTTTATTT
Pda_00005807_2R	TAGGGGAGCAACAGCAAATT
Pda_00005807_3F	ACGGCTGGGCAGCTGGCTGGCT
Pda_00005807_3R	AGTAAAAAGTCAATTGTTTA
Pda_00005807_4F	TGGACCCGAGCCTGATCTTATT
Pda_00005807_4R	TTTTATTGGGCGGGATTTTT
Pda_00034448_1F	AGGTATTTTTATATTAAATCAT
Pda_00034448_1R	ACTAAATAGATATTTTATTT
Pda_00034448_2F	TTTTTCATCGAAAATTTTCATA
Pda_00034448_2R	TGGATGTTCTTTAATTTTAG
Pda_00034448_3F	AGACTTGCGTCGATTCCTCCTTT
Pda_00034448_3R	AAAACCACACACACAGCGGC
Pda_00034448_4F	TGTTTTTTAATTTGAAAGTTATTG
Pda_00034448_4R	TATTAATTAAACTGACCAGA
Pda_00026132_1F	TAAAACATTGATATCCAAACGA
Pda_00026132_1R	TATTGTATCATTATTTCGTA
Pda_00026132_2F	TTGACGATGCCATGAGTGCAAG
Pda_00026132_2R	CATCTCGTAATTTTGATAGT
Pda_00026132_3F	ATTAGTCAAGTTTCTGAATGATA
Pda_00026132_3R	ATATGTTTGAATTTGTGTAA
Pda_00026132_4F	CTTCTTTGATTCTAATAATTCAA
Pda_00026132_4R	TGCTATGGGTATAGCATGCT

Primer names	Primer sequences (5'-3')
Pda_00032259_1F	GGTATCACTGAGGACTTTGAT
Pda_00032259_1R	GGAGCACATGTAATAGAAAT
Pda_00032259_2F	CCCTCTTTAGCATTTGCCTCTTT
Pda_00032259_2R	TAATTTAATTATATTATCAT
Pda_00032259_3F	TTAAATAATTACTTATGTCTGAA
Pda_00032259_3R	TTTACCTTTTAATTAAAACT
Pda_00032259_4F	GAAAAAAAATTAATTTTTTATT
Pda_00032259_4R	TTTGGGCTTTTATATTTTTT
Pda_00021056_1F	TCAATTTTTTAATTTGTAAGATT
Pda_00021056_1R	CTAAAACCAAAAAAAAAAAA
Pda_00021056_2F	ATGATTTTAACATGTTATGTTAA
Pda_00021056_2R	TATGACGAAGTTAAAAATCT
Pda_00021056_3F	TCCTTCAGCCACTTGATAGCAC
Pda_00021056_3R	TTCTTATTCTCCTCTAATAA
Pda_00021056_4F	TTATTTAACCAAATAGAAAATAT
Pda_00021056_4R	TTGCTTGCTCTCTTAGCAAC
Pda_00021677_1F	GAATAGAGAGGCATAGCCATTAA
Pda_00021677_1R	GCATGAGTGAAAAGATGCAT
Pda_00021677_2F	TTGAAAAAAATGTGGCTTGCAA
Pda_00021677_2R	CAAAGAAAAATCGATCCATT
Pda_00021677_3F	CATTTTCCGTGAGCACAAAGCA
Pda_00021677_3R	GCTCTCTCTTCGGGTACTTG
Pda_00021677_4F	TTGGTAGCTAATATGAAATTAA
Pda_00021677_4R	GTTTCTCTATTTTGGCAAAG
Pda_00013035_1F	GCCTCTGGTGAAGGTCTTTCTAA
Pda_00013035_1R	TGCATCCATCATATCTTTCC
Pda_00013035_2F	TTTGTCAAGTACTACAAGTAC
Pda_00013035_2R	TATAAAATAAAAATTCAACC
Pda_00013035_3F	AGAATTTTCTATTATTTATGC
Pda_00013035_3R	TTCATATTTGACATAGAAAA
Pda_00013035_4F	ATTACTACGTGTATGTTTGTTT
Pda_00013035_4R	GATCGATAATATGAACCCGA
Pda_00041854_1F	CGTCATTGATAGTGCCCCTCTTC
Pda_00041854_1R	CTAACGTGGAAATAATTTAA
Pda_00041854_2F	AGTTAAAATCTGAGAAAGGT
Pda_00041854_2R	TCTTGTAGCCAGATTATTAT
Pda_00041854_3F	AAAGAGGGTTCGATGGAGGAAG
Pda_00041854_3R	TAAAAAGGTCAAGTGTTCTT
Pda_00041854_4F	ATTAAAAAGCTTACACATGAC
Pda_00041854_4R	TTGTTATTGTTTGTGTTGAT

Primer names	Primer sequences (5’-3’)
Pda_00016418_1F	TCTGAGCTTGGAATTTTTTTTTT
Pda_00016418_1R	AAATCATACCTACCTCTTTA
Pda_00016418_2F	ATCTATGATTTTTTCTTGATTTT
Pda_00016418_2R	TAAAATCAAGAAAAAAAATG
Pda_00016418_3F	TCCTTCAATTTTGGATTGGTTC
Pda_00016418_3R	CACTAAATTATGCTGAAAAA
Pda_00016418_4F	GAAGTTCACTCTCTTTAGACAA
Pda_00016418_4R	TTTCTTTCGATCTCCTTGGG
Pda_00035137_1F	TAAAATGAAACATTTATTACAC
Pda_00035137_1R	CCAGCCAACGCAGGAAAGAA
Pda_00035137_2F	TTCTATTGTTTTCTCTTTTTTGTT
Pda_00035137_2R	AAGTGACTTAATCGTACAAC
Pda_00035137_3F	TCAATCAACTTTTTATTAAAA
Pda_00035137_3R	AAAACAATATCAACATATAA
Pda_00035137_4F	TAATTGTTTAGTTTATTTGATG
Pda_00035137_4R	CAGGCGGGAGAACGAACAAC
Pda_00009408_1F	ATTGATCTTCAGAAATTATCATG
Pda_00009408_1R	CAAAAAATTTAATTTTTAGC
Pda_00009408_2F	TGGTTTGATTCGTTTTGGTAC
Pda_00009408_2R	TTGTCCTGGCTGGTTTCATC
Pda_00009408_3F	ATTATTGTTAGATTGATTGTTA
Pda_00009408_3R	TGTTTTTTTGTCAGTCAGGG
Pda_00009408_4F	CGGCAAAAATAACCCCTCAAAA
Pda_00009408_4R	TATTTGAAAAACGAAAAACA
Pda_00029282_1F	TGACCGTGTGTTCCAGTTTGTG
Pda_00029282_1R	TTATTATTATAGTTTTTAGT
Pda_00029282_2F	AATATTTAAATTAACATAGTT
Pda_00029282_2R	AAAATTAGATTATTTATAAT
Pda_00029282_3F	TTTTGTTGATTTTATTATAACA
Pda_00029282_3R	TAATTTTTTTAAAAATCCAT
Pda_00029282_4F	AAAGAAAAAAACATTATTGG
Pda_00029282_4R	CATTTTGATTGGGAAAAGAC
Pda_00022335_1F	CAGCAGCCCAAGCATCTCCT
Pda_00022335_1R	TATGCTTCCCCCCCTTTTCA
Pda_00022335_2F	CCTTAAAGTGTCGGCAAACGTT
Pda_00022335_2R	CTAACGATGGTAAGGAACCT
Pda_00022335_3F	TTGGGTGGCATCTTGTCATTC
Pda_00022335_3R	TGGAATTTGCTTTCCAAGAA
Pda_00022335_4F	TTACCAAACTCTACTTTTTTTC
Pda_00022335_4R	TATTCTTAAGATTTCACCCT
