# Supplementary material for: Construction of a Hierarchical Gene Regulatory Network to Reveal the Drought Tolerance Mechanism of Shanxin Poplar
Source: Int J Mol Sci. 2022 Dec 26;24(1):384. doi: 10.3390/ijms24010384 (PMC9820611; doi:10.3390/ijms24010384)
Supplement: Supplementary file 1 [file ijms-24-00384-s001.zip › Table S9 .pdf]

Table S9. The primers used for qRT-PCR.

Primer names	Primer sequences (5’-3’)
Pda_00023176_qF	CCAATAGGCTGTCCCTGCTTAAA
Pda_00023176_qR	GTCTGCAGATTAGGCTTCTGCATTTG
Pda_00010869_qF	AGACACTCCGATGGAATC
Pda_00010869_qR	GGGTATACTACTGGATAAG
Pda_00019195_qF	TAAACTTCTCGAGCTTATTGG
Pda_00019195_qR	CTCCCTGATTGCTCCAAA
Pda_00034144_qF	AGCTTTTGCTATCCAACGAG
Pda_00034144_qR	GAAGCAGCAGATTGTTTC
Pda_00025305_qF	GGTGGAGAACCTTGCTC
Pda_00025305_qR	TCTTTCAGGTCATGAGC
Pda_00039367_qF	GTGACTTCTCCAGTATCCAAGCC
Pda_00039367_qR	TGAGATGTATGTGGAAGTTG
Pda_00037432_qF	CACATGGAGGAGTGTATGCA
Pda_00037432_qR	TGCACTCTCAGCATCACCATTG
Pda_00006818_qF	TGCCACTGCTGAAAGCGTC
Pda_00006818_qR	CAACAATGTCCTCAAGTTGA
Pda_00031022_qF	AATCACCCGAACGTTGGTGC
Pda_00031022_qR	CATCCACAGTGAACGTGTC
Pda_00036271_qF	AATGGAGGGGAAGGAAGGG
Pda_00036271_qR	CAACTGACAAAGGCACGG
Pda_00000970_qF	GTTTAGTCGGAGATCGATTGCCG
Pda_00000970_qR	AAGCAGCCTGCGGTCCAATT
Pda_00002376_qF	AGAATAAGTTACAGAGTG
Pda_00002376_qR	AAAAATATCTGGACCT
Pda_00003683_qF	TGGATTTGAGCTTGGCT
Pda_00003683_qR	ATGTTCCTCGGCAGCTTT
Pda_00006007_qF	CTTCCCGCACCAGCACAA
Pda_00006007_qR	TGTTGCATCTCGAATGGC
Pda_00022729_qF	GGTATGATGCGGAGAAGATA
Pda_00022729_qR	TCACCAGCAATGTGTGCTT
Pda_00016968_qF	ATGCCATTTGGGGGAGG
Pda_00016968_qR	TTATATGACTGTGTGGTGCT
Pda_00008662_qF	GTTCATGCTTGACAAGATCG
Pda_00008662_qR	TACGGCATGTACTTGCT
Pda_00036802_qF	TATGCTGCAGCAAATTTGTC
Pda_00036802_qR	CATACATCTGCGATCT
Pda_00040672_qF	TTCCCATCTCTTCATTCGG
Pda_00040672_qR	GTACTCACGACGAACTGC
Pda_00002023_qF	TATTGGAATGTTATAGTTTG
Pda_00002023_qR	CCCGGCCGCAATTGTCCT

Primer names	Primer sequences (5’-3’)
Pda_00011485_qF	GGTCAAGGAGTTGAGATTGTTAG
Pda_00011485_qR	GCAATTGCAAGGCATGAAGTA
Pda_00028118_qF	GGCCCAACAGCCAGTA
Pda_00028118_qR	TCCTCATATACCCACT
Pda_00022832_qF	AAGTACGACTTCTCTCCTTT
Pda_00022832_qR	AAAGAAATCAGTATCATG
Pda_00012337_qF	AGCAGCAGAGGGGGGCTAG
Pda_00012337_qR	GACAGTTGGTAAGGTCAA
Pda_00019030_qF	ATCTAGCAATTGGTGCCATAT
Pda_00019030_qR	GAAGTTGCGAACAAAC
Pda_00005807_qF	TCAACAGTGTTGTCGGGG
Pda_00005807_qR	GACCGCCTTTTCG
Pda_00034448_qF	CAGGGAAGGAAGAAAAG
Pda_00034448_qR	CTGCTCACTTCCCTGC
Pda_00026132_qF	GTATCAGAATATACGAGGA
Pda_00026132_qR	CTATCGATGAAATCTTGCAATT
Pda_00021677_qF	GTCAGCACCCTTTGCCCAAA
Pda_00021677_qR	CATGAACCATGCCATCAC
Pda_00013035_qF	TTTCTTCGGAGGAGATCAGATCGG
Pda_00013035_qR	TGGCAAACTCAACAAGCTTC
Pda_00041854_qF	AGGATGACTCTGCTCTTAGGA
Pda_00041854_qR	TCTAATGTTTCTCTCACA
Pda_00009408_qF	CTAAGAGGAAATCAGGCTCAA
Pda_00009408_qR	TCCACCACCGCAACCTCCG
Pda_00029282_qF	AGAGCCTCCAACTCCCCGTTCA
Pda_00029282_qR	TCCAACAGCACTGAGAGCAT
Pda_00022335_qF	ATAGAGGTGCATCTATCCCTAAG
Pda_00022335_qR	CTGCAACTAATCCTGCATTT
Pda_00032259_qF	ATTACTATACTGCATATTATG
Pda_00032259_qR	TCTTAACGACTTCACCGTAG
Pda_00003956_qF	GTGTATTCACATTTGCGAGAT
Pda_00003956_qR	CAAAATATGCCGGCACTCCA
Pda_00007115_qF	CAAATAATCCTGCGTTATTGG
Pda_00007115_qR	CTGTTATAAACCCAATTGATC
