# Supplementary figures and images for: Construction of a Hierarchical Gene Regulatory Network to Reveal the Drought Tolerance Mechanism of Shanxin Poplar
Source: Int J Mol Sci. 2022 Dec 26;24(1):384. doi: 10.3390/ijms24010384 (PMC9820611; doi:10.3390/ijms24010384)

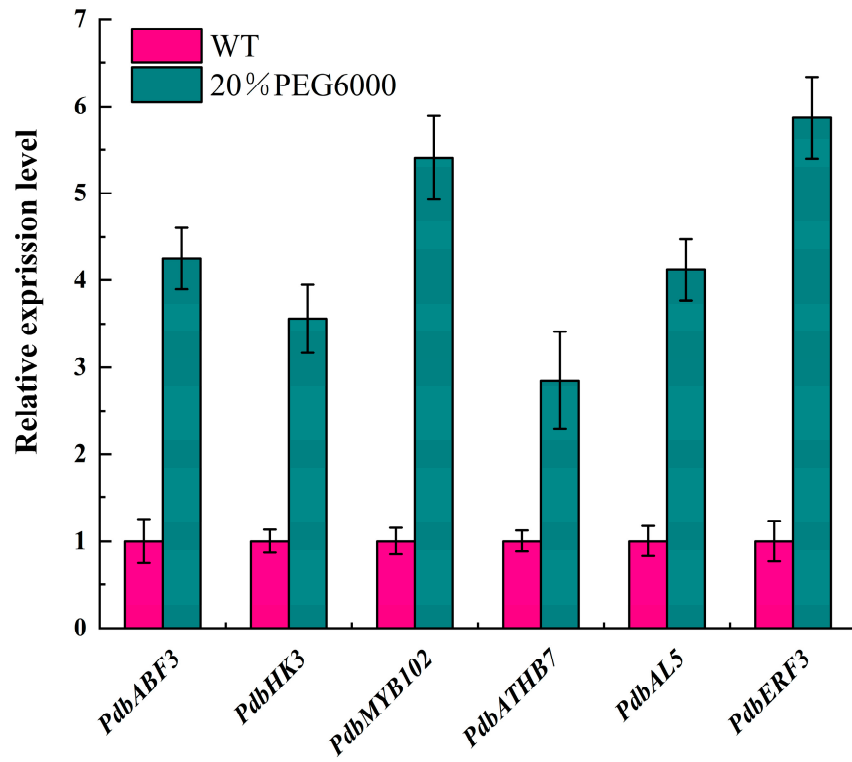

**Figure S3. qRT-PCR verify the expression of related genes in first-layer TFs under drought stress.**

Supplement: Supplementary file 1 [file ijms-24-00384-s001.zip › Figure S3.pdf]
